# Chemoselective Characterization of New Extracellular Matrix Deposition in Bioengineered Tumor Tissues

**DOI:** 10.1002/adma.202505445

**Published:** 2025-09-06

**Authors:** Zihan Ling, Burke Niego, Qingyang Li, Dhruv Bhattaram, Vanessa Serna Villa, Michael Hu, Zhuowei Gong, Lloyd M. Smith, Brian L. Frey, Xi Ren

**Affiliations:** ^1^ Department of Biomedical Engineering Carnegie Mellon University Pittsburgh PA 15213 USA; ^2^ Department of Chemistry University of Wisconsin Madison WI 53706 USA

**Keywords:** biomaterial, chemoselective chemistry, newly‐synthesized extracellular matrix, proteomics

## Abstract

In both native and engineered tissues, the extracellular matrix (ECM) supports and regulates nearly all aspects of cellular pathophysiology, and in response, cells extensively remodel their surrounding extracellular environments through new ECM protein deposition. Understanding this intricate bi‐directional cell‐ECM interaction is key to tissue engineering, but it remains challenging to investigate. This is partly due to the limited sensitivity of conventional proteomics to capture low‐abundance newly synthesized ECM (newsECM). This study presents a glycosylation‐enabled, chemoselective strategy to label, enrich, and characterize newsECM proteins with augmented specificity and sensitivity. Applying newsECM profiling to bioengineered tumor tissues, either built upon decellularized ECM materials (dECM‐tumor) or as ECM‐free tumoroids, revealed distinct ECM synthesis patterns. Tumor cells cultured within dECM scaffold present elevated ECM remodeling activities, mediated by augmented digestion of pre‐existing ECM coupled with upregulated synthesis of tumor‐associated ECM components. These findings highlight the sensitivity of newsECM profiling to capture remodeling events that are otherwise under‐represented by bulk proteomics and underscore the significance of dECM support for enabling native‐like tumor cell behaviors. The newsECM profiling described here is anticipated to be applicable to a wide range of engineered tissue models and pathophysiological processes to deliver fundamental insights regarding the mutual cell‐ECM crosstalk.

## Graphical abstract

Understanding the bi‐directional crosstalk between cells and their surrounding extracellular matrix (ECM) is critical for tissue engineering. This study presents a chemoselective strategy for proteomic profiling of newly deposited ECM from resident cells. Applying this analytical pipeline to bioengineered tumor tissues reveals elevated ECM remodeling activities in tumor cells in response to biomaterial support and the associated culture conditions.

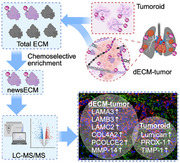

## 1 Introduction

The extracellular matrix (ECM) is a complex meshwork of proteins and glycosaminoglycans that not only provides structural support to resident cells but also acts as a signaling pool, delivering critical microenvironmental cues to regulate cellular pathophysiology^[^ ^1^, ^2^ ^]^ Contemporaneously, the interaction between the ECM and cells is not unidirectional. Instead of remaining static, the ECM undergoes dynamic remodeling by resident cells through processes such as deposition, modification, and degradation.^[^ ^3^ ^]^ Frequently, ECM remodeling is observed to progress in synergy with cellular transformation to mediate both physiological tissue morphogenesis and pathological fibrogenesis and tumorigenesis.^[^ ^3^, ^4^ ^]^ A key mechanism for cells to remodel their surrounding ECM is through regulated secretion of new ECM components—in particular proteins—into the extracellular space to either make structural contributions to the formation of new ECM structures or modify/degrade the pre‐existing ECM.^[^ ^5^ ^]^ Thus, capturing and understanding the dynamic synthesis and deposition of new ECM proteins is essential to elucidate how resident cells respond to their evolving cellular and extracellular states in various pathophysiological contexts.

Tumorigenesis is characterized by somatic cell transformation into a state characterized by uncontrolled proliferation, with a further potential to invade and spread into other tissues.^[^ ^6^, ^7^, ^8^ ^]^ The unique ECM properties of solid tumors have long been underscored by elevated stiffness and density compared to healthy tissue counterparts, favoring cancer cell survival and expansion. The acquisition of these tumor‐specific ECM features is a result of continuous ECM remodeling by tumor cells.^[^ ^9^ ^]^ These cells secrete proteases such as matrix metalloproteinases (MMPs) that degrade the original benign tissue ECM and replace it with tumor‐specific ECM.^[^ ^9^ ^]^ Tumor cells also release growth factors and other signaling proteins to the extracellular niche to promote angiogenesis and metastasis.^[^ ^8^ ^]^

Bioengineered 3D tissues have emerged as powerful model systems for recapitulating disease processes, investigating pathogenic mechanisms, and developing new therapeutics.^[^ ^10^, ^11^, ^12^ ^]^ A common strategy for tissue engineering is to combine cells with ECM materials that deliver the desired biochemical composition, biomechanical property, and/or architecture of the tissue‐specific extracellular microenvironment—or a combination of these aspects, as occurs with decellularized native organ scaffolds.^[^ ^13^ ^]^ Nonetheless, it is also observed that in the absence of exogenous ECM support, many cell types, such as benign epithelial cells and tumor cells, have the intrinsic ability to self‐assemble into organotypic structures in the form of spheroids or organoids,^[^ ^14^, ^15^ ^]^ where the extracellular architecture is formed solely by *de novo* ECM synthesis and deposition by the constituent cells. How do cells remodel the ECM materials that they are attached to, and how does the presence of pre‐existing ECM or biomaterial support modulate the ECM secretome of cells? These fundamental questions in tissue engineering are yet to be fully understood.

A key challenge in studying ECM‐modulation of new ECM production in the context of tissue engineering is how to differentiate new versus pre‐existing ECM proteins. Stable isotopic labeling of amino acids in cell culture^[^ ^16^ ^]^ has enabled the proteomic detection of new protein synthesis through the incorporation of heavy‐isotope amino acids.^[^ ^17^ ^]^ However, mass spectrometry‐based proteomics is an abundance‐proportional technique that favors identifying high‐abundance protein species, but it falls short in capturing subtle changes of low‐abundance proteins, many of which play key regulatory roles in altering the ECM microenvironment through direct (crosslinking, chemical modification, and degradation) and indirect (modulating cellular behavior and their ECM secretome) mechanisms.^[^ ^18^ ^]^ Other technologies, such as RNA sequencing, only allow access to the instantaneous ECM gene expression at the transcriptomic level.^[^ ^19^ ^]^ Therefore, there is a need for proteomics‐based technologies that reveal critical information related to translational and post‐translational control, which ultimately determines the quantity, stability, and biochemical properties of newly deposited ECM proteins.^[^ ^20^, ^21^ ^]^

One method of post‐translational control highly prevalent in ECM and ECM‐associated proteins is glycosylation. Glycosylation is one of the most common types of post‐translational modifications (PTMs) where polysaccharide side chains are added to the newly synthesized core proteins. Glycosylation takes place primarily in the endoplasmic reticulum and Golgi apparatus, organelles that synthesize, modify, and transport proteins destined for secretion into the extracellular space. Therefore, glycosylation is highly prevalent in ECM and ECM‐associated proteins that are mostly secreted;^[^ ^22^ ^]^ in fact, it is estimated to occur for more than 90% of secreted proteins in humans.^[^ ^23^, ^24^, ^25^ ^]^ Consistent with this, our prior work has established the high efficacy of bioorthogonal labeling of new ECM deposition using azido‐tag‐bearing monosaccharide probes both in vivo and ex vivo, with azido‐galactosamine tetra‐acetylated (Ac_4_GalNAz) being the most effective.^[^ ^26^, ^27^ ^]^ These azido‐tagged ECM proteins are compatible with chemoselective conjugation via click chemistry with a wide variety of ligands that enable a broad spectrum of downstream applications such as ECM material functionalization.^[^ ^28^ ^]^

In this study, we navigated bioorthogonal ECM labeling to a new direction for ECM discovery and utilized a chemoselective strategy to profile newly synthesized ECM (referred to as newsECM) produced by resident cells and secreted onto the decellularized ECM (dECM) scaffolds. Using a bioengineered lung cancer model combining tumor cells and a whole‐lung dECM scaffold, we demonstrated effective labeling, detection, enrichment, and proteomic identification of newsECM glycoproteins. Leveraging this robust analytical pipeline, we characterized and compared the newsECM profiles within tumor tissues derived from the same tumor cells formed with and without exogenous ECM scaffolds. Our comparison revealed elevated ECM remodeling activities of the tumor cells when cultured within dECM scaffolds. We anticipate the presented technology to be widely applicable for investigating cell‐ECM interaction and delineating how pre‐existing biomaterial ECM and the associated culture conditions regulate ECM remodeling in the context of tissue engineering.

## 2 Main Text

### 2.1 Engineering 3D Tumor Tissues in the Presence and Absence of dECM Support

**Figure 1:**
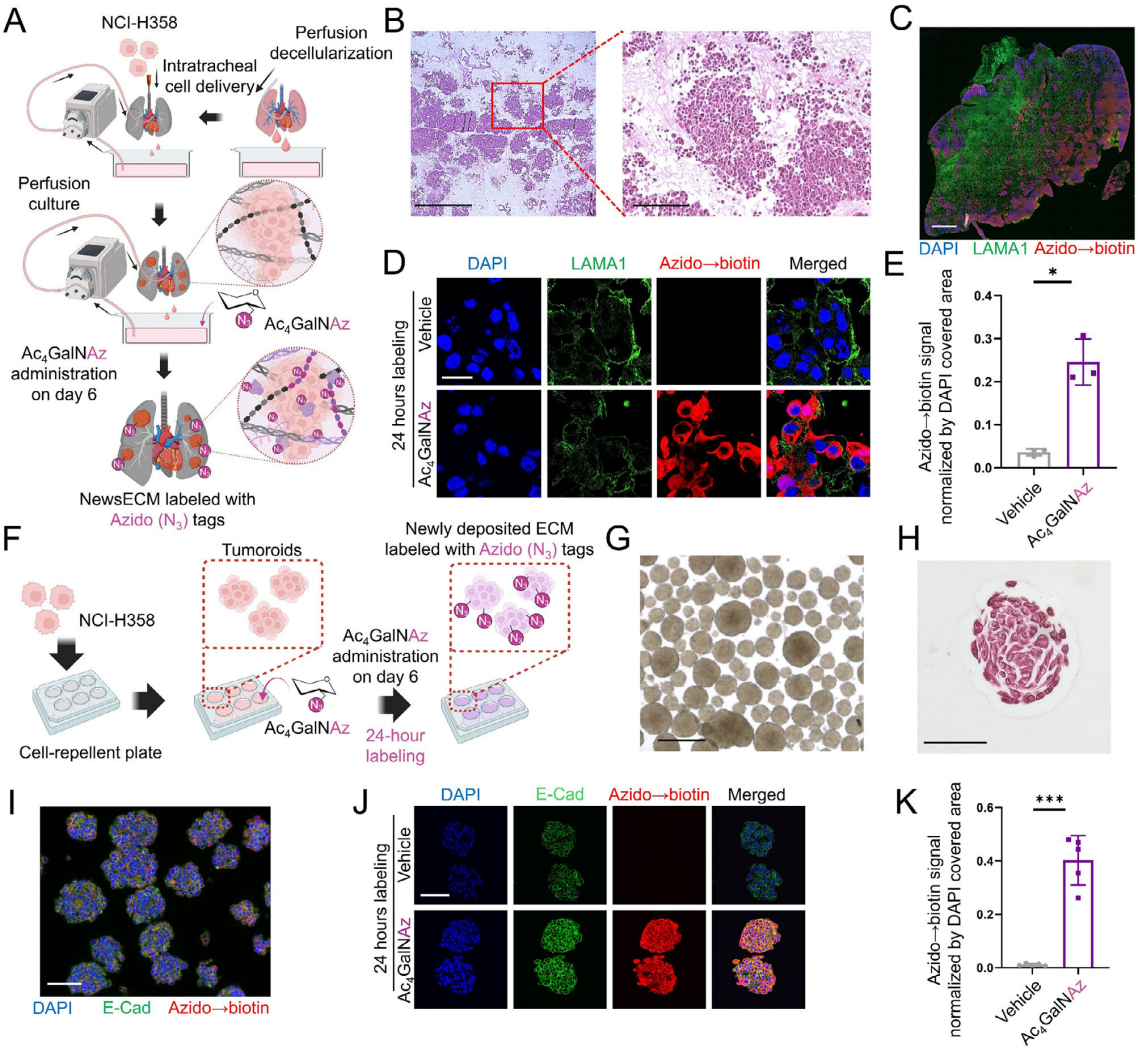
Glycosylation‐enabled metabolic labeling of newsECM in engineered tumor models. A) A schematic for the engineering of dECM‐tumor model and metabolic newsECM labeling using Ac_4_GalNAz. B) Hematoxylin and eosin (H&E) staining of the dECM‐tumor after 7 days of perfusion culture. Scale bars: left, 1000 µm; right, 200 µm. C,D) Stitched image (C) or single‐field images (D) of immunofluorescence staining of azido→biotin (red) and LAMA1 (green) on dECM‐tumors receiving Ac_4_GalNAz (*n* = 3) or DMSO (vehicle control, *n* = 3) during the last day of culture. Azido tags were visualized with fluorophore‐conjugated streptavidin detection of biotin following alkyne‐PEG4‐biotin conjugation. Scale bar in panel C, 1000 µm. Scale bar in panel D, 30 µm. E) Quantification of azido→biotin (red) signal intensities in panel D, normalized by DAPI covered areas. The image fields selected for quantification are demonstrated in Figure S23 (Supporting Information). The data was analyzed by two‐tailed *t*‐test with Welch's correction. F) A schematic for generating the ECM‐free tumoroid model and metabolic newsECM labeling using Ac_4_GalNAz. G) Bright‐field image of tumoroids at the end of 7‐day culture. Scale bar, 500 µm. H) H&E staining of tumoroids following 7 days of agitation culture. Scale bar: 75 µm. I, J) Low‐magnification image (I) or high‐magnification images (J) of immunofluorescence staining of azido→biotin (red) and E‐Cadherin (E‐Cad, green) on tumoroid clusters receiving Ac_4_GalNAz (*n* = 5) or DMSO (vehicle control, *n* = 5) during the last day of culture. Scale bars in both panel I and panel J, 100 µm. K) Quantification of azido→biotin (red) signal intensities in panel J, normalized by DAPI covered areas. The data was analyzed by two‐tailed *t*‐test with Welch's correction. *** *p*<0.001; * *p*<0.05. Data are presented as means ± SD. Schematics were created with Biorender.com and published with permission.

Tumor cells are well known to extensively alter their surrounding extracellular microenvironment to favor their expansion and metastasis.^[^ ^9^ ^]^ To model and investigate these key pathological processes, 3D tumor tissues can be engineered in vitro either with or without exogenous biomaterial support.^[^ ^29^, ^30^ ^]^ Yet our understanding of how the biomaterial environment modulates the ability of tumor cells to produce new ECM, along with the properties of these ECM depositions, remains elusive. To investigate this, we established two 3D tumor tissue models using NCI‐H358 lung cancer cells, either as cell‐ECM composites or as ECM‐free tumor spheroids (tumoroids). To engineer the cell‐ECM composites, we leveraged dECM scaffolds prepared from perfusion decellularization of whole rat lungs and seeded NCI‐H358 cells through intratracheal instillation into what was the epithelial compartment prior to decellularization (**Figure** **1**A).^[^ ^29^ ^]^ To better capture the ECM architectures of regions where tumor tissues developed, we first performed histological analysis for this composite of tumor cells and dECM scaffold at 1 day following intratracheal cell delivery, to facilitate recognition of ECM architectures prior to profound ECM remodeling by tumor cells taking place. Whole‐lobe scan of tumor cell‐seeded dECM scaffold (Figure S1, Supporting Information) suggests tumor cell distribution across the entire scaffold with more cells located along the periphery. Close‐up examination of randomly selected scaffold regions populated by tumor cells revealed an overall ECM architecture that appeared interconnected and polyhedral‐shaped (Figure S1, Supporting Information), indicative of distal (alveolar) regions of the lung. With perfusion culture through the well‐preserved native pulmonary vascular bed,^[^ ^29^, ^31^ ^]^ the seeded tumor cells effectively engrafted onto the dECM material, leading to formation of tumor node clusters by day 7 of culture (Figure 1B). These resulting engineered tumor tissues on dECM scaffolds were referred to as “dECM‐tumors”. In parallel, we generated ECM‐free tumoroids over the same culture duration through the self‐assembly of cancer cells on a cell‐repellent surface coupled with constant agitation (Figure 1F–H).

### 2.2 Glycosylation‐Enabled Bioorthogonal Labeling of newsECM Deposition in the dECM‐Tumor and ECM‐Free Tumoroid

To selectively study new ECM protein deposition by resident cells within an ECM biomaterial environment, such as the decellularized lung, we implemented a bioorthogonal labeling strategy that allows distinguishing newsECM proteins from the pre‐existing ones. Our strategy utilized an azido‐tagged monosaccharide probe, Ac_4_GalNAz, that can be specifically incorporated into the glycans of newly synthesized glycoproteins, including those secreted into the extracellular space, during post‐translational glycosylation.^[^ ^23^, ^24^, ^25^ ^]^ To apply this labeling procedure to the dECM‐tumor model, we administered Ac_4_GalNAz (or dimethyl sulfoxide (DMSO) as vehicle control) into the medium on day 6 of tumor tissue culture for a 1‐day labeling period (Figure 1A). The azido‐tag incorporation was visualized through click conjugation with alkyne‐biotin followed by fluorescence detection of biotin. We observed robust azido‐tag incorporation within the dECM‐tumor tissues, showing specific colocalization with regions of tumor nodule presence (Figure 1C–E). To exclude the possibility of azido tagging through passive labeling of the dECM scaffold in the absence of tumor cell involvement, we administered the Ac_4_GalNAz probe to a dECM lung scaffold without cell seeding for the same 1‐day labeling duration, which led to undetectable level of azido probe incorporation (Figure S2, Supporting Information). Of note, the main source of LAMA1 (Laminin Subunit Alpha 1) signal detected was from the dECM scaffold and thus it was present throughout the scaffold, in regions both with and without the presence of tumor cells (DAPI) (Figure 1D; Figure S2, Supporting Information). These findings together support the requirement of cellular metabolism for azido labeling of newly synthesized glycoproteins. To allow subsequent comparison of newsECM profiles between dECM‐tumors and ECM‐free tumoroids, we further validated that the same Ac_4_GalNAz labeling procedure is applicable to tumoroids cultured in the same medium, leading to specific azido‐tag incorporation throughout the tumoroids (Figure 1F,I–K).

### 2.3 ECM Extraction and Chemoselective Enrichment of newsECM

**Figure 2:**
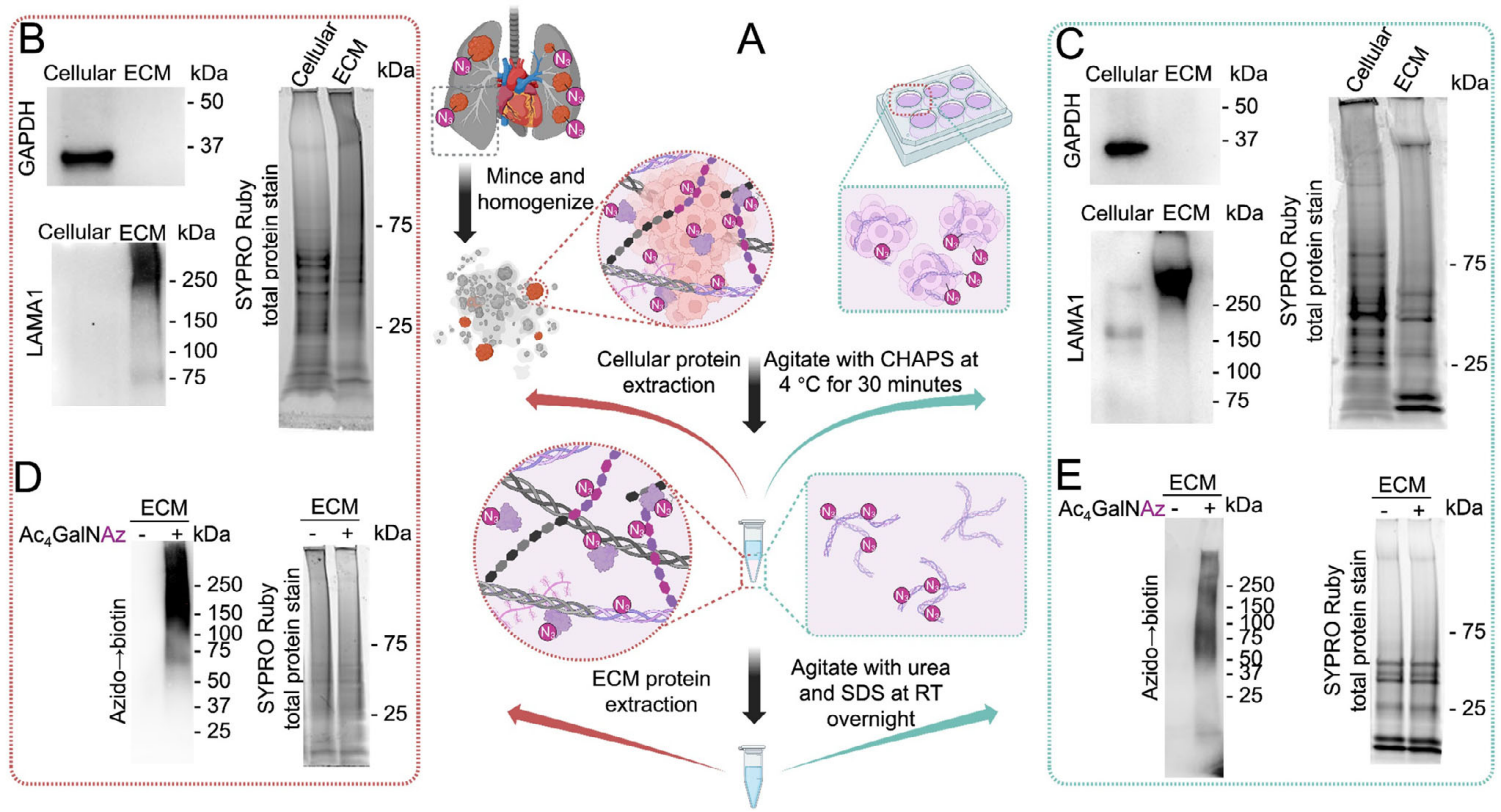
Stepwise extraction of cellular and ECM fractions in bioengineered tumor models. A) A schematic for stepwise extraction of cellular and ECM proteins from dECM‐tumors (left) and tumoroids (right). B,C) Western blot detection of GAPDH (left, top) and LAMA1 (left, bottom) and SYPRO Ruby staining of total proteins (right) in cellular and ECM fractions from dECM‐tumors (B) and tumoroids (C). D,E) Western blot detection of azido→biotin signal in the ECM fractions of dECM‐tumors (D) and tumoroids (E) using streptavidin‐HRP (left) and SYPRO Ruby staining of total proteins (right). Schematics were created with Biorender.com and published with permission.

While the ECM is particularly enriched for glycoproteins, many cellular proteins are also glycosylated. To focus our analysis on the ECM, we developed a stepwise extraction method to isolate and enrich the extracellular protein contents. We first extracted the cellular protein fraction with a mild 3‐[(3‐cholamidopropyl) dimethylammonio]‐1‐propanesulfonate (CHAPS) buffer, followed by extraction of the extracellular protein fraction with a harsher urea‐based buffer (**Figure** **2**A). The extraction efficiency and specificity were validated by western blot analysis of cellular GAPDH (Glyceraldehyde 3‐phosphate dehydrogenase) and TUBA1A (Tubulin alpha‐1a chain) or extracellular LAMA1 (Laminin Subunit Alpha 1) and Fibronectin (FN). We observed nearly exclusive presence of GAPDH and LAMA1 protein signals to the cellular and extracellular fractions, respectively, in both dECM‐tumors and ECM‐free tumoroids (Figure 2B,C left), with equal protein loading confirmed by SYPRO Ruby total protein stain (Figure 2B,C right). This was further confirmed by the analysis of TUBA1A and Fibronectin in tumoroid extracts (Figure S3A–C, Supporting Information). Upon validating our stepwise protein extraction, we went on to examine azido labeling in each protein fraction via alkyne‐biotin conjugation and on‐blot biotin detection. Consistent with prior tissue staining findings (Figure 1D,J), specific azido signals were detected in both cellular and ECM fractions of both dECM‐tumors and tumoroids administered with Ac_4_GalNAz compared to those administered with vehicle control (Figures S4 and S5, Supporting Information; Figure 2D,E). To further confirm the molecular specificity of Ac_4_GalNAz labeling of newsECM, we performed a competition labeling assay on tumoroids by co‐administering Ac_4_GalNAz together with excessive competing N‐Acetyl‐D‐galactosamine (GalNAc), which led to significant reduction in azido labeling in tumoroid ECM (Figure S6, Supporting Information), suggesting azido incorporation via the GalNAc salvage pathway.

**Figure 3:**
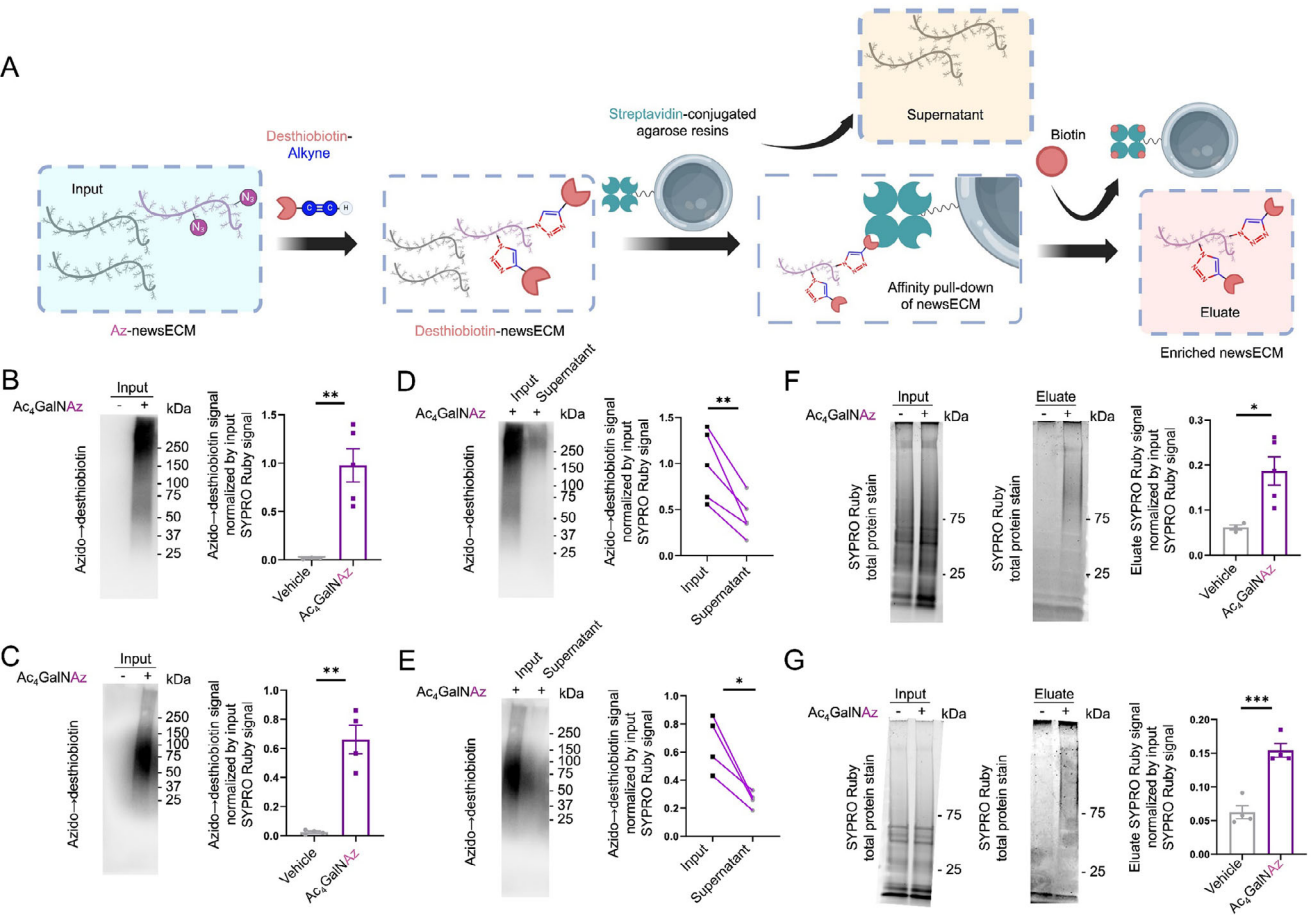
Chemoselective enrichment of azido‐newsECM. A) A schematic showing the workflow for chemoselective enrichment of Az‐newsECM. The Az‐newsECM were desthiobiotinylated (input), pulled down by streptavidin resins (the wash‐offs were saved as supernatants), and released through competitive elution with high‐affinity biotin (eluates). B,C) Western blot analysis (left) and quantification (right) of desthiobiotin signal in the ECM inputs of dECM‐tumors (B) and tumoroids (C) receiving Ac_4_GalNAz or DMSO (vehicle control) using streptavidin‐HRP. The data was analyzed by two‐tailed *t*‐tests with Welch's correction. D,E) Western blot analysis (left) and quantification (right) of desthiobiotin signal in the ECM inputs and supernatants of dECM‐tumors (D) and tumoroids (E) receiving Ac_4_GalNAz using streptavidin‐HRP. The data was analyzed by paired *t*‐tests. F,G) SYPRO Ruby staining of total proteins in the ECM inputs (left) and eluates (middle) of dECM‐tumors (F) and tumoroids (G), and quantification (right) of eluate SYPRO Ruby signals. The data was analyzed by two‐tailed *t*‐tests with Welch's correction. All signal quantifications were normalized by their corresponding input SYPRO Ruby signals. *** *p*<0.001. **p<0.01, *p<0.05. Data are presented as means ± SD. Schematics were created with Biorender.com and published with permission.

To chemoselectively purify the azido‐tagged newsECM (Az‐newsECM) from the total extracted ECM, we converted azido labeling into desthiobiotin tags through click conjugation with alkyne‐desthiobiotin, followed by streptavidin pull‐down^[^ ^27^ ^]^ (**Figure** **3**A). We first demonstrated specific alkyne‐desthiobiotin conjugation onto Az‐newsECM in both dECM‐tumors and ECM‐free tumoroids with prior Ac_4_GalNAz administration (Figure 3B,C). Upon introducing the desthiobiotinylated ECM samples to streptavidin resins, we analyzed the azido→desthiobiotin signals from samples collected both before (“input”) and after (“supernatant”) incubation with streptavidin resins and observed a dramatic reduction in azido→desthiobiotin signals in the supernatants compared to inputs (Figure 3D,E), demonstrating high efficacy of streptavidin resins to capture desthiobiotinylated newsECM. To examine protein capturing specificity, we pre‐blocked streptavidin resins with free biotin (Figure S7A, Supporting Information), which led to minimal resin‐binding of desthiobiotinylated newsECM proteins as indicated by similar azido→desthiobiotin signals comparing input and supernatant samples (Figure S7B–D, Supporting Information), validating the high selectivity of streptavidin pull‐down. Finally, we released the captured newsECM proteins through competitive elution using free biotin with higher affinity to streptavidin compared to desthiobiotin^[^ ^32^ ^]^ (Figure 3A). SYPRO Ruby total protein stain confirmed specific protein elution from ECM inputs of Ac_4_GalNAz‐labeled dECM‐tumors and tumoroids, compared to those from tissues administered with vehicle control (Figure 3F,G). Altogether, these results demonstrate effective and specific enrichment of Az‐newsECM from engineered tumor tissues for subsequent proteomics analysis.

### 2.4 Proteomics Characterization of newsECM Signatures

**Figure 4:**
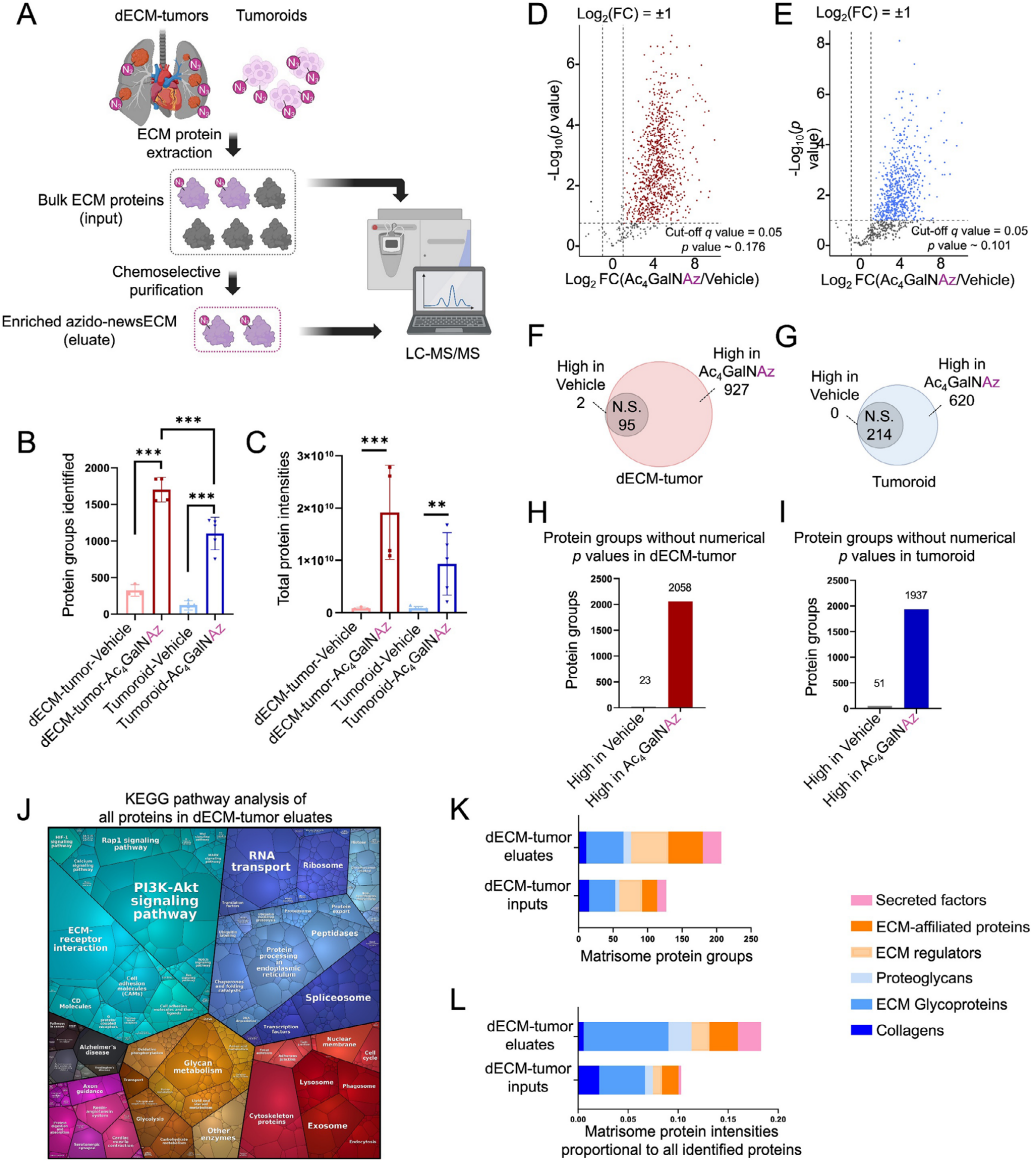
Proteomics characterization and Gene Ontology (GO) analysis of newsECM profiles in bioengineered tumor models. A) A schematic of the workflow for proteomics characterization of newsECM in dECM‐tumors and tumoroids. B) A bar graph plotting the protein groups identified in eluate samples from dECM‐tumors receiving Ac_4_GalNAz (*n* = 5) or DMSO (vehicle control, *n* = 3) or tumoroids receiving Ac_4_GalNAz (*n* = 5) or DMSO (vehicle control, *n* = 5). Outliers were identified and cleared out by Grubbs's tests (*α* = 0.05). Data was analyzed with one‐way ANOVA with Tukey's multiple comparisons tests (confidence level: 0.95). C) A bar graph plotting the total protein intensities (before normalization) identified in eluate samples from dECM‐tumors receiving Ac_4_GalNAz (*n* = 4) or DMSO (vehicle control, *n* = 3) or tumoroids receiving Ac_4_GalNAz (*n* = 5) or DMSO (vehicle control, *n* = 5). Data was analyzed with one‐way ANOVA with Tukey's multiple comparisons tests (confidence level: 0.95). (D‐I) Differential comparisons of protein intensities between Ac_4_GalNAz and DMSO (vehicle control) eluates from dECM‐tumors (D,F,H) or tumoroids (E,G,I). Protein groups with comparisons that cannot be assigned with numerical *p* values (due to having fewer than 2 nonzero values in either treatment group) were pre‐filtered out and plotted as bar graphs based on their average intensities in each treatment group (H,I). D) Volcano plot for proteins identified in eluate samples from dECM‐tumors receiving Ac_4_GalNAz (*n* = 4) or DMSO (vehicle control, *n* = 3). The proteins fall into three categories: high in Ac_4_GalNAz (red, Log_2_FC>1, *q*<0.05), high in Vehicle (black, Log_2_FC<−1, *q*<0.05) and non‐significant (N.S., gray, *q*≥0.05 or ‐1≤Log_2_FC≤1). E) Volcano plot for proteins identified in eluate samples from tumoroids receiving Ac_4_GalNAz (*n* = 5) or DMSO (vehicle control, *n* = 5). The proteins fall into three categories: high in Ac_4_GalNAz (blue, Log_2_FC>1, *q*<0.05), high in Vehicle (black, Log_2_FC<−1, *q*<0.05) and non‐significant (N.S., gray, *q*≥0.05 or ‐1≤Log_2_FC≤1). For both panel D and E, the decimal intensities were transformed into Log_2_ and analyzed with two‐tailed *t*‐tests with Welch's correction, followed by multiple hypothesis testing correction with permutation‐based FDR (FDR < 0.05) to compute *q* values. FCs were computed as fold changes of geometric means. The *p* value cut‐off lines were plotted based on *q* = 0.05 (*p* ∼ 0.176 in panel D; *p* ∼ 0.101 in panel E). F,G) Venn diagrams showing the distribution among the three categories in panel D or panel E. J) A KEGG pathway Proteomap generated with all eluate proteins from the dECM‐tumor receiving Ac_4_GalNAz. The area of each pathway represents its enrichment level and the color‐coding represents different categories. K,L) Matrisome analysis of inputs and eluates from dECM‐tumors. The numbers (K) or total intensities (L) of proteins identified for each matrisome category were plotted for both inputs and eluates of the dECM‐tumors. *** *p*<0.001; ** *p*<0.01. Data are presented as means ± SD. Schematics were created with Biorender.com and published with permission.

To characterize and compare newsECM signatures in both tumor models, we performed LC‐MS/MS analysis on the ECM samples both before (“inputs”) and after (“eluates”) the streptavidin pull‐down (**Figure** **4**A). To further support our stepwise protein extraction protocol for focused analysis of the ECM, we surveyed the abundances of major intracellular components (actins, tubulins, and histones) in both dECM‐tumor and tumoroid inputs, and found that they together accounted for less than 1% of the total input protein intensities (Table S1, Supporting Information, 0.36% in dECM‐tumor and 0.81% in tumoroid). Through the analysis of the eluate proteomes prefiltered for human proteins, we observed significant augmentation in both the identified protein groups (5.3 fold in dECM‐tumors and 9.0 fold in tumoroids) and total protein intensities (23.4 fold in dECM‐tumors and 11.0 fold in tumoroids) in eluates from tumor tissues with Ac_4_GalNAz labeling compared to eluates from tissues exposed to vehicle control (Figure 4B,C). This observation is consistent with protein abundance results shown in the volcano plot comparisons (Figure 4D,E), Venn diagrams (Figure 4F,G), and bar charts (Figure 4H,I), and is further in line with the total protein stain findings shown previously (Figure 3F,G). These results together validate our approaches described here for chemoselective newsECM enrichment. Since the dECM‐tumors were constructed combining human cells with dECM derived from rat lungs, we anticipate the newsECM profiling to be particularly enriched for human proteins that were directly labeled through NCI‐H358 metabolism. To assess this, we normalized human‐ or rat‐specific proteins identified in eluates based on their relative intensities in the inputs and observed that while human protein intensities detected in eluates remained at a level close to those of the inputs (87.5% of inputs), rat proteins in eluates were reduced to 30% of inputs (Figure S8, Supporting Information). The remaining rat proteins identified (1.2% of total eluate protein intensities) were likely captured through indirect mechanisms such as interaction with the resin‐bound Az‐newsECM. This further supports the selectivity of the described newsECM profiling toward new protein production by metabolically active cells. To eliminate the influence from proteins of rat origin, we filtered our eluate proteomic data for human proteins in subsequent analyses (Table S4, Supporting Information).

### 2.5 Gene Ontology (GO) Analysis of newsECM Profiles from dECM‐Tumors

Upon validating newsECM enrichment at the proteomics level, we went on to investigate the newsECM protein deposition patterns starting with the dECM‐tumors. We first enriched the top Kyoto Encyclopedia of Genes and Genomes (KEGG) pathways from the dECM‐tumor eluates based on the quantitative composition of each individual protein.^[^ ^33^ ^]^ The most active KEGG pathways linked to newsECM production in the dECM‐tumors are associated with PI3K‐Akt signaling and ECM‐receptor interaction (Figure 4J). This supports the biological relevance of the newsECM profiling, as the PI3K‐Akt cascade and the pathways associated with cell‐substrate adhesion are frequently overactivated in native tumor tissues.^[^ ^34^ ^]^ The abundance plot of individual proteins (Figure S9, Supporting Information) also shows higher abundances of ECM structural proteins such as Laminins (e.g., Laminin Alpha‐3, Gamma‐2 and Beta‐3 subunits), Tenascin‐C (TNC), Fibronectin 1 (FN1), and Heparan Sulfate Proteoglycan 2 (HSPG2), as well as ECM/cell surface signaling proteins such as Integrins (e.g., Integrin Beta‐1, Alpha‐6 and Beta‐4), suggesting that the tumor cells are actively depositing new structural ECM components onto the pre‐existing dECM scaffold to reinforce interactions with their extracellular environment. The GO functional annotation clustering^[^ ^35^, ^36^ ^]^ of the top 100 abundant proteins in dECM‐tumor newsECM also enriched terms with high biological relevance, including “ECM‐receptor interaction”, “proteoglycans in cancer”, and “extracellular matrix” (Table S2, Supporting Information). To further categorize the revealed matrisomal protein synthesis, we matched the proteins identified in newsECM (eluates) and bulk ECM (inputs) from dECM‐tumors to a human matrisome dataset.^[^ ^37^ ^]^ Our results suggest that compared to bulk proteomics, newsECM profiling identified more matrisome protein groups in general, especially ECM regulators (21 more), ECM‐affiliated proteins (28 more), and secreted factors (14 more) (Figure 4K). We also plotted the matrisome protein intensities in proportion to all identified proteins in eluates or inputs and observed that while newsECM profiling detected less scaffolding proteins like collagens (26% of those found in inputs), it identified higher intensities of other matrisome proteins such as ECM regulators (2.0 fold of those found in inputs), proteoglycans (2.9 fold of those found in inputs), and secreted factors (9.2 fold of those found in inputs) (Figure 4L). These analyses suggest that our approach, by selectively profiling newsECM, avoids the high background signals generated by pre‐existing structural proteins within the biomaterial scaffold and offers an augmented capability to detect low‐abundance extracellular species involved in ECM remodeling.

### 2.6 Differential Analysis Reveals Distinct newsECM Patterns Regulated by dECM Scaffold Culture

**Figure 5:**
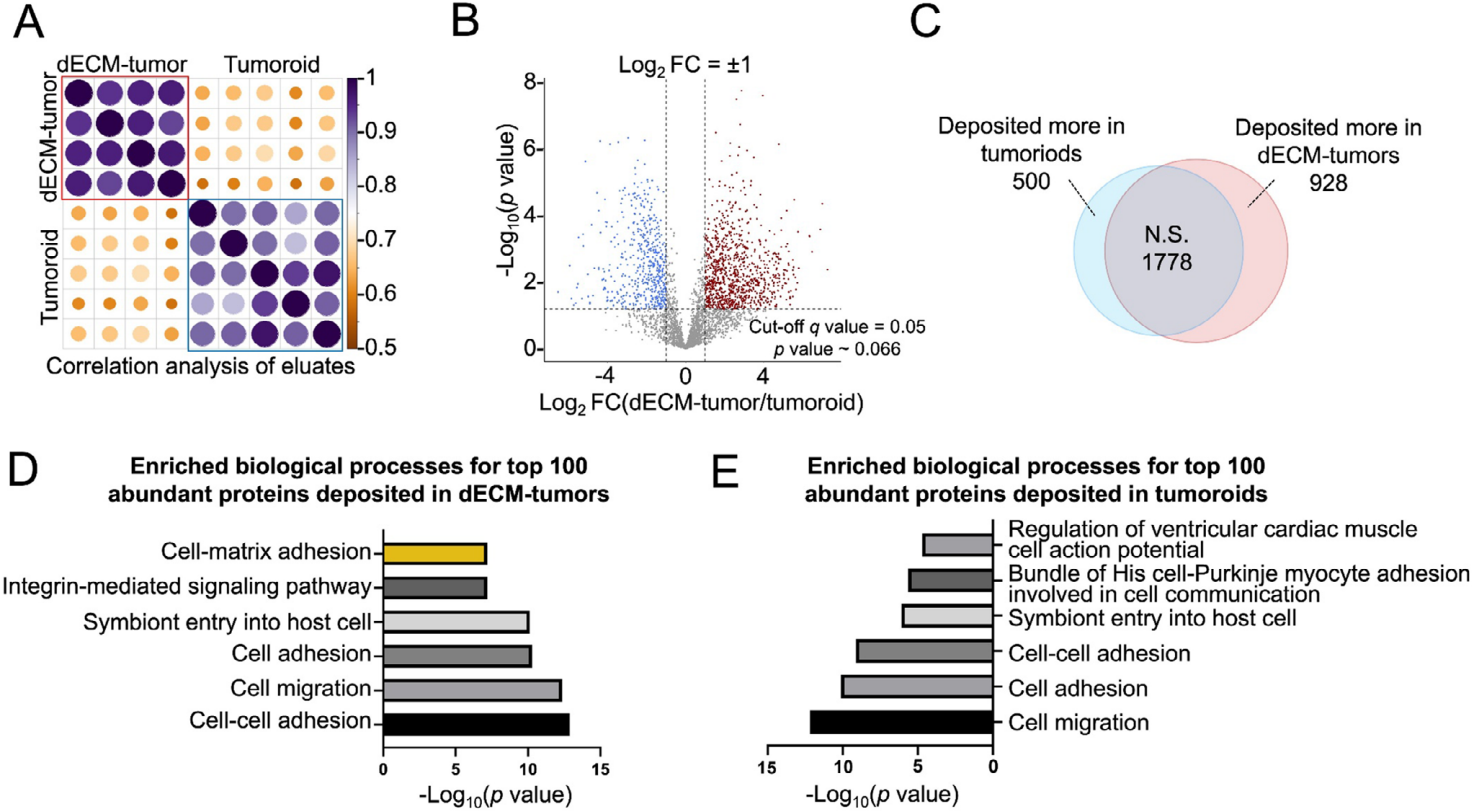
Differential analysis shows distinct newsECM patterns regulated by dECM scaffold. A) Correlation dot plot for proteomic profiles of eluates samples from dECM‐tumors (*n* = 4) and tumoroids (*n* = 5) receiving Ac_4_GalNAz. Pearson correlation analysis was performed between each pair of samples. The colors indicate correlation levels, and the dot sizes indicate the significance of the correlation. Purple, high; Orange, low. Large, significant; Small, non‐significant. B) Volcano plot of proteins identified in eluate samples from dECM‐tumors (*n* = 4) or tumoroids (*n* = 5) receiving Ac_4_GalNAz. The decimal intensities were transformed into Log_2_, imputed based on a quantile regression imputation of left centered missing data (QRILC) method and analyzed with two‐tailed *t*‐test with Welch's correction, followed by multiple hypothesis testing correction with permutation‐based FDR (FDR < 0.05) to compute *q* values. The proteins fall into three categories: high in dECM tumors (red, Log_2_FC>1, *q*<0.05), high in tumoroids (blue, Log_2_FC<−1, *q*<0.05) and non‐significant (N.S., gray, *q*≥0.05 or ‐1≤Log_2_FC≤1). FCs were computed as fold changes of geometric means. The *p* value cut‐off line was plotted based on *q* = 0.05 (*p* ∼ 0.066). C) Venn diagram showing the distribution among three categories in panel B. (D,E) GO analysis of top enriched biological process terms of the 100 proteins with highest intensities in eluates samples from dECM‐tumor (D) or tumoroid (E) receiving Ac_4_GalNAz.

To compare the differences in ECM synthesis patterns of tumor cells when cultured with or without dECM scaffold support, we compared the newsECM profiles between dECM‐tumors and ECM‐free tumoroids. The correlation dot plot shows high correlation between replicates within each tissue model and low correlation between the replicates from two different models (**Figure** **5**A), suggesting high consistency of the newsECM profiling as well as distinct newsECM profiles influenced by the dECM scaffold and the associated culture conditions. This is further supported by scatter plots of individual protein intensities showing high correlation (R^2^ ∼ 0.94, Figure S10A,B, Supporting Information) between replicates from the same tissue model and low correlation (R^2^ ∼ 0.49, Figure S9C, Supporting Information) between replicates from different models. The volcano plot (Figure 5B) and Venn diagram (Figure 5C) show the number of extracellular proteins with differential synthesis levels between the two models: 928 and 500 proteins with upregulated synthesis in dECM‐tumors and ECM‐free tumoroids, respectively. We enriched the GO “biological processes” terms for the top 100 abundant proteins from the newsECM in each model (Figure 5D,E) and identified similar top enriched GO terms for both tumor models, except for “cell‐matrix adhesion” which was specifically highlighted in dECM‐tumors. In order to avoid the interference from cytoplasmic protein contaminants in newsECM profiles, we also analyzed the “Cellular Component” GO terms (GO:CC) of these top 100 abundant proteins and observed enrichment of GO:CC terms closely related to the extracellular space and cell surface (Figure S11A,B, Supporting Information). In addition, we filtered the eluate proteomes for proteins found in the extracellular space based on their GO tags in Swiss‐Prot.^[^ ^38^ ^]^ The enriched “biological processes” terms for the top 100 abundant extracellular proteins from the newsECM in each model (Figure S12A,B, Supporting Information) suggest similar findings, further validating our observations. Together our analyses suggest that the tumor cells can sense and adapt to their surrounding environment by tailoring their ECM secretome.

### 2.7 Elevated ECM Digestive Activities Associated with dECM Scaffold Culture

**Figure 6:**
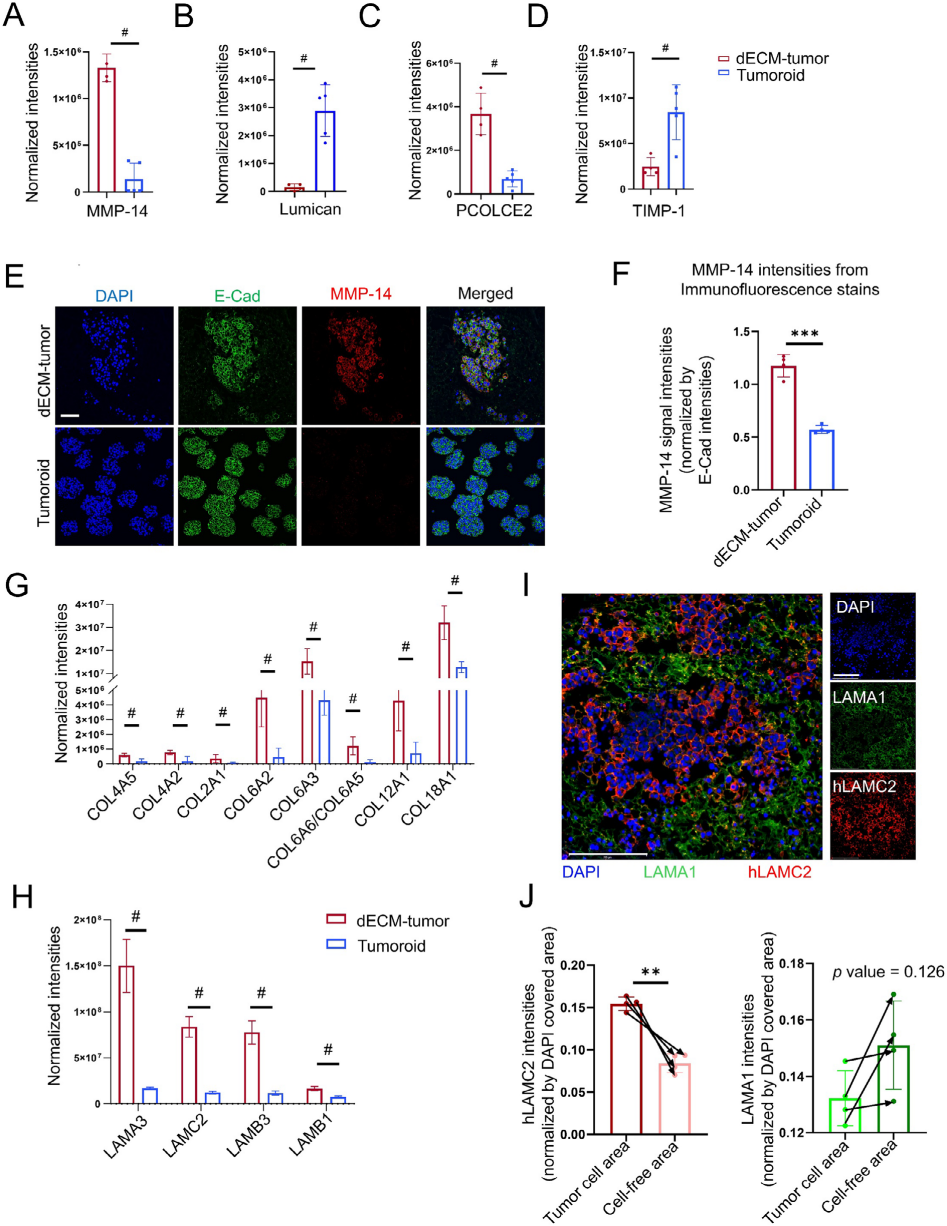
Tumor cells presented elevated remodeling activities when cultured within dECM scaffold. A–D) Bar graph showing the normalized, imputed protein intensities of MMP‐14 (A), Lumican (B), PCOLCE2 (C), and TIMP‐1 (D) in eluate samples from dECM‐tumors (left, red, *n* = 4) and tumoroids (right, blue, *n* = 5) receiving Ac_4_GalNAz. The significance was determined by FDR‐corrected *q* value. E) Immunofluorescence staining of MMP‐14 (red) and E‐Cad (green) in dECM‐tumors (*n* = 4) and tumoroids (*n* = 4). Scale bar: 50 µm. F) Quantification of MMP‐14 signal intensities in areas populated with cells (based on DAPI signal) in panel E, normalized by E‐Cad intensities. The data was analyzed by two‐tailed *t*‐tests with Welch's correction. G,H) Bar graphs showing the normalized, imputed protein intensities of Collagen (G) or Laminin (H) subunits in eluate samples from dECM‐tumors (red, *n* = 4) or tumoroids (blue, *n* = 5) receiving Ac_4_GalNAz. The significance was determined by FDR‐corrected *q* values. I) Immunofluorescence staining of LAMA1 (green) and hLAMC2 (red) in dECM‐tumors (*n* = 4). Scale bar: 275 µm. J) Bar graphs quantifying LAMA1 and hLAMC2 signal intensities in panel I. Each image was separated into two parts based on DAPI signal: areas populated with cells (with DAPI) and cell‐free areas (without DAPI). The hLAMC2 (left) and LAMA1 (right) signals on each area were plotted in the bar graphs. Arrows show data point connections for paired signal intensities from the same areas in the same images. The data was analyzed by paired *t*‐tests. ^#^ *q*<0.05; *** *p*<0.001; ** *p*<0.01. Data are presented as means ± SD. Zero values (when the protein was not observed by MS proteomics) were replaced with imputed values as described in Methods (Proteomic Data Analysis).

Tumor cells have long been recognized to actively degrade and remodel the ECM from the surrounding benign tissue environment to facilitate tumor expansion and invasion.^[^ ^9^ ^]^ Laminins are crucial basement membrane components of the lung that play essential roles to support luminal cells while limiting apical‐to‐basolateral cell migration.^[^ ^39^ ^]^ Intriguingly, immunofluorescence analysis of Laminin distribution in the dECM‐tumor tissues revealed a reduction in LAMA1 abundance specifically within regions populated by the tumor nodes (Figure S2, Supporting Information). Consistent with this finding, we identified a series of selective changes in the newsECM secretome of dECM‐tumors that were indicative of augmented ECM digestive activities, including upregulation of ECM degrading enzymes and downregulation of their inhibitors. For example, we observed Membrane‐Type Matrix Metalloproteinase I (MT‐MMP1, also known as MMP‐14) with significantly elevated synthesis in dECM‐tumors compared to ECM‐free tumoroids (**Figure** **6**A). This supports the biological relevance of our tissue models and newsECM profiling, as overexpression of MMP14 is frequently associated with excessive tumor ECM remodeling, migration, and metastasis.^[^ ^40^, ^41^ ^]^ In parallel to eluates, we also searched the bulk proteomics results of input samples from both dECM‐tumors and tumoroids, and were not able to detect MMP‐14, further demonstrating the augmented sensitivity of newsECM profiling to identify subtle changes in low‐abundance ECM regulators compared to conventional bulk proteomics. In line with these findings, our newsECM profiling also identified lower synthesis levels of Lumican, a recently identified MMP‐14 inhibitor,^[^ ^42^ ^]^ in dECM‐tumors compared to tumoroids (Figure 6B). In addition to MMP‐14, other ECM digestive regulators, such as Procollagen C‐Endopeptidase Enhancer 2 (PCOLCE2), a predictive marker for epithelial‐to‐mesenchymal transition and metastasis in lung cancer,^[^ ^43^ ^]^ were also observed with elevated synthesis levels in dECM‐tumors (Figure 6C). In contrast, MMP inhibitors, such as Tissue Inhibitor of Metalloproteinase 1 (TIMP‐1), were found with lower deposition levels in dECM‐tumors (Figure 6D). Together these newsECM profiles suggest elevated matrix degradation activities in the dECM‐tumors.

To validate the proteomics findings above, taking MMP‐14 as an example we performed immunofluorescence staining in both tumor tissue types. We observed significantly elevated MMP‐14 presence colocalizing with tumor cell colonization in dECM‐tumors in comparison to tumoroids (Figure 6E,F), which was localized both intracellularly and at the cell surface, shown by co‐localization with E‐cadherin (E‐Cad) (Figure S13, Supporting Information). The findings above were further supported by MMP‐14 western blot analysis on ECM extracts from both tumor models (Figure S14, Supporting Information). To investigate the possibility of loss of secreted MMP‐14 fragments into the tumoroid culture medium, we also probed the medium and observed undetectable level of MMP‐14, similar to our finding from tumoroid ECM, which together support the augmented MMP‐14 deposition in dECM‐tumors compared to tumoroids (Figure S15, Supporting Information). These findings further support the authenticity of our newsECM profiling approach and demonstrated that tumor cells presented higher ECM‐digestive activities when exposed to the dECM scaffold and associated culture conditions.

### 2.8 Augmented Tumor ECM Deposition Linked to dECM Scaffold Culture

In parallel to aggressively digesting the surrounding benign tissue environments, tumor cells are also well known to actively lay down their own ECM species to build a more favorable extracellular environment for themselves to thrive.^[^ ^9^ ^]^ Comparing the newsECM profiles between the two tumor models, we observed enhanced deposition of structural ECM components including several collagen and laminin subunits in dECM‐tumors compared to tumoroids (Figure 6G,H). Notably, we identified significantly higher deposition levels of Laminin Alpha‐3, Beta‐3 and Gamma‐2 subunits in dECM‐tumors (Figure 6H), which are the subunits of Laminin‐5 (Laminin‐332) with reported roles in tumor progression and migration.^[^ ^44^ ^]^ To validate this finding, we performed immunofluorescence staining against one of the tumor‐associated laminins (human Laminin Subunit Gamma 2, hLAMC2) in dECM‐tumor tissue sections and observed its expression to be concentrated at the extracellular space in close vicinity to the tumor cells (Figure S16, Supporting Information, Figure 6I,J left). Interestingly, we observed a reduction in LAMA1 (which is an important component of the basement membrane in healthy lungs)^[^ ^45^, ^46^ ^]^ at tumor cell loci (Figure 6I,J right), suggesting that the tumor cells within the dECM‐tumors exert a synergistic influence to remodel their extracellular environment through combined ECM‐digestive and synthetic activities. Taken together, our newsECM profiling establishes a comprehensive understanding regarding the ECM deposition landscape in bioengineered tumor models and how it is being modulated by the surrounding ECM environment, biomaterial support, and associated culture conditions.

## 3 Conclusions and Discussion

The ECM is a key constituent in nearly all native tissues and is accordingly being used extensively as scaffold material in the field of tissue engineering for both disease modeling and regenerative medicine applications.^[^ ^11^ ^]^ Despite the wide recognition of the bidirectional nature of cell‐ECM interactions, our understanding remains limited regarding how the ECM and biomaterial support regulates cells’ ability to remodel their extracellular environment. Previous studies have established robust technologies to metabolically label newsECM with azido‐bearing monosaccharides,^[^ ^47^ ^]^ chemoselectively functionalize azido‐tagged proteins with click chemistry,^[^ ^48^ ^]^ and proteomic ECM characterization with label‐free LC‐MS/MS.^[^ ^49^ ^]^ In this study, we repurposed and combined these approaches to deliver a complete analytical pipeline for chemoselective profiling of new ECM deposition by resident cells into their surrounding biomaterial ECM. We showcased the application of this analytical pipeline to two synthetic tumor tissue models with distinct extracellular environments. Comparing the newsECM proteomic profiles between the dECM‐tumor and ECM‐free tumoroid models, we uncovered a profound influence of the dECM scaffold and associated culture conditions on the ECM secretome of resident tumor cells. This observation was highlighted by elevated ECM remodeling by the dECM‐exposed tumor cells, mediated by augmented digestion of pre‐existing ECM coupled with upregulated synthesis of tumor‐associated ECM. We anticipate the technological platform described here to facilitate future inquiries regarding how cells respond to their surrounding biomaterial environments in a wide variety of tissue engineering scenarios as well as ECM remodeling events taking place in vivo.

Tumor cells are well‐known for their ability to build an extracellular environment that favors their own growth and enhances their plasticity for metastasis.^[^ ^9^ ^]^ By comparing the newsECM profiles between two tumor models in which cells were cultured within or without dECM scaffold, we found that the exposure to biomaterial scaffold supported culture directed the tumor cells to adopt an ECM deposition pattern that better recapitulates what is observed in native tumor tissues. For example, we detected with our newsECM proteomics analysis and observed augmented synthesis in dECM‐tumors of MMP‐14, one of the reported driving forces behind ECM destruction during tumor cell invasion.^[^ ^40^, ^41^, ^50^, ^51^ ^]^ Our immunofluorescence staining reveals MMP‐14 localization both intracellularly and at the cell surface in dECM‐tumors (Figure S13, Supporting Information), indicating that the tumor cells present active synthesis and trafficking of MMP‐14. In line with this, we also observed reduced deposition levels of Lumican (MMP‐14 inhibitor) in dECM‐tumors (Figure 6B). Additionally, a recent clinical study correlated upregulated MMP‐14 with reduced Prospero Homeobox Protein 1 (PROX‐1),^[^ ^52^ ^]^ which is identified as a transcriptional repressor of MMP‐14.^[^ ^53^ ^]^ Consistent with this, reduced PROX‐1 synthesis was also captured in dECM‐tumors compared to tumoroids in our study (Figure S17, Supporting Information). Concurrent with elevated secretion of ECM‐digesting enzymes in dECM‐tumors, we observed specific reduction of LAMA1 signals at the tumor cell loci. Laminins are mandatory trimers, where the trimerization of its alpha, beta and gamma subunits takes place intracellularly following their translation and prior to secretion, thus the degradation of LAMA1 observed in dECM‐tumors is likely coupled with the digestion of the associated laminin beta and gamma chains.^[^ ^54^ ^]^

It is important to note that although we observed an intriguing concurrence of increased secretion level of MMP‐14 with elevated basement membrane digestive activity, we are not yet able to establish direct causal relationships between the observed events. Protein‐protein interaction network functional enrichment analysis with the MatrixDB database showed that MMP‐14 physically interacts with other critical ECM degrading enzymes such as MMP‐9 (Figure S18, Supporting Information). In fact, MMP‐14 plays an important role in activating MMP‐9^[^ ^55^ ^]^ that is involved in degrading basement membrane proteins such as Collagen IV.^[^ ^56^ ^]^ Consistent with this, we observed a higher synthesis level of TIMP‐1, an MMP‐9 inhibitor,^[^ ^57^, ^58^ ^]^ in the tumoroids compared to dECM‐tumors (Figure 6D). In addition to MMP‐14, we also observed elevated deposition of other pro‐digestive ECM regulators, such as PCOLCE2, in dECM‐tumors (Figure 6C). Together these proteomic signatures support the enhanced ECM degradation activities of tumor cells regulated by biomaterial scaffold derived from decellularized lungs. However, extensive future studies, such as the use of cells with controlled MMP‐14 expression, will be required to address the direct effects of MMP‐14 on dECM scaffolds.

In parallel to the discovery of newsECM associated with matrix degradation, we also observed augmented deposition of tumor‐associated basement membrane components in dECM‐tumors compared to that in tumoroids. For example, in dECM‐tumors we observed upregulated synthesis of all three subunits of Laminin‐332 (Figure 6H,I) that is closely associated with aggressive tumor invasion,^[^ ^44^, ^59^, ^60^ ^]^ with the Gamma 2 subunit being reported as a therapeutic target against tumor aggravation.^[^ ^61^ ^]^ In addition to Laminins, in dECM‐tumors we also observed elevated deposition of Alpha‐2 Chain of Type IV Collagen (COL4A2, Figure 6G), another major component of the tumor microenvironment with potential involvement in the pathogenesis and progression of multiple cancer types, including lung cancer.^[^ ^62^ ^]^ Collectively, our results suggest that the incorporation of the dECM scaffold and associated culture conditions tunes the tumor ECM deposition toward patterns with closer resemblance to those found in native tumors, and that investigating the cellular ECM secretome can be an effective way to assess pathophysiological relevance of bioengineered tissue models.

Glycosylation is a common PTM with frequent occurrence in proteins being secreted to the extracellular space (predicted to be over 90% in humans).^[^ ^23^, ^24^, ^25^ ^]^ Therefore, bioorthogonal labeling via glycosylation has the potential of tagging a significant portion of newly synthesized and deposited ECM proteins. The chemoselective newsECM profiling approach described here enriches extracellular proteins newly deposited by resident cells over a defined culture period, free from the pre‐existing ones of the biomaterial scaffold, enabling their selective characterization with improved detection sensitivity. In addition, our approach also avoids the proteomics background generated by high‐abundance structural ECM proteins (such as collagens) and allows more focused detection of ECM species with signaling and regulatory roles which are often of low abundances. For example, we were able to identify MMP‐14 as a molecular signature with high consistency in dECM‐tumor newsECM eluates (Figure 6A) but were unable to detect it in the inputs from the same tissue samples with conventional bulk proteomics. In addition, the relatively low turnover rates of structural ECM proteins lead to their accumulation over time within bioengineered tissues,^[^ ^63^ ^]^ thus masking the detection with regular mass spectrometry of signaling proteins that undergo more dynamic turnover. The described newsECM profiling offers a strategy to overcome this challenge and enables study of the dynamics of low‐abundance ECM regulators that may be under‐investigated in traditional proteomics. Consistent with our study, a recent report pioneered the labeling and visualization of nascent matrix proteins produced by chondrocytes within polymer hydrogels by directly targeting new peptide synthesis using an azido‐bearing methionine analog (L‐azidohomoalanine)^[^ ^64^ ^]^ This study together with ours highlights the versatility of click chemistry‐based strategies for probing cellular remodeling of their surrounding matrix environment.

Proteomic analysis of input protein samples validated our stepwise extraction method to remove the majority of cytosolic proteins from the ECM fraction, and very low level of actins, tubulins and histones can be detected (Table S1, Supporting Information). In the meantime, our method preserves and detects plasma membrane‐associated proteins with direct interaction with ECM processes (such as the membrane‐bound MMP‐14), offering a unique opportunity to analyze the deposition of molecules that mediate the crosstalk between cells and their surrounding matrix. Notably, we analyzed the top 100 abundant proteins in both dECM‐tumor and tumoroid inputs and enriched for GO:CC terms. Our results suggest extracellular matrix, cell surface or plasma membrane as top enriched CC terms for inputs from both models (Figure S19A,B, Supporting Information), further supporting that our approach enriches for proteins in the extracellular space.

We acknowledge that our newsECM profiling method bears some limitations. Analysis of ECM proteins can be a challenging process given the varying solubility of different components, and the numerous PTMs these proteins bear that can further complicate extraction, solubility, and downstream analysis.^[^ ^65^ ^]^ Since a key focus of our investigation is signaling molecules that facilitate cell‐ECM interactions, we applied a gentle extraction buffer condition (8mm CHAPS with 1m NaCl) for removing cellular contents prior to ECM extraction. Such strategy helped retain not only the ECM proteins, but also membrane proteins interacting or associated with the matrix, such as MMP‐14, for the extraction and detection in newsECM profiles. In addition, we also applied a less harsh extraction buffer (4m urea with 0.1% sodium dodecyl sulfate (SDS)) for the ECM fraction compared to the commonly used buffer conditions,^[^ ^66^ ^]^ leaving behind insoluble ECM pellets that represent aggregated or crosslinked ECM components and are beyond our analysis. As a result, certain collagen subunits, as well as elastin, may not have been sufficiently solubilized and extracted for proteomic detection. For example, collagens commonly have a trimeric organization, but not all participating subunits were recovered in our analysis (Figure 6G). If understanding these low‐solubility ECM components is of interest, harsher extraction conditions such as chemical digestion (for example, using hydroxylamine)^[^ ^67^ ^]^ can be employed to enhance ECM protein extraction yield. Moreover, the newsECM protein labeling and profiling strategy described here depends on their glycosylation. Despite the high prevalence of protein glycosylation in the extracellular space, our approach will inevitably miss non‐glycosylated proteins that may also take part in ECM remodeling. Other targeted proteomics approaches such as bioorthogonal non‐canonical amino acid tagging (BONCAT)^[^ ^68^ ^]^ that label newly synthesized proteins by directly targeting their amino acids may offer insights regarding these non‐glycosylated proteins. Future comparison between the glycan‐ and amino acid‐targeted proteomics profiling will be helpful to provide better understanding regarding their potential differences in terms of protein coverage, labeling efficacy, and detection sensitivity.

In this study, we compared the newsECM proteomes between the dECM‐tumor and tumoroid models and observed elevated remodeling activity of tumor cells when cultured within the dECM scaffold. Notably, this effect can be attributed to a combination of factors, including dECM composition, scaffold architecture, spatial confinement of cells, as well as diffusion dynamics of nutrients and wastes. Future investigations, such as controlled manipulation of dECM composition or culture dynamics, will be necessary to tease out the contribution of each of these contributing factors to the regulation of tumor cell ECM secretome. Furthermore, it remains unclear whether the observed changes in newsECM secretome become intrinsic to these tumor cells and to what extent such secretome will be maintained once the cells are removed from the dECM scaffold culture. Future incorporation of ECM removal and recovery assays may provide critical insights in this regard; while such assay can be challenging to perform in the dECM scaffold with complex architectures, it may be more feasible in contexts where the biomaterial supports can be easily dissociated from cells, such as in hydrogel‐embedded tissue cultures.^[^ ^69^ ^]^ Moreover, extending Ac_4_GalNAz metabolic labeling period beyond 24 h can be a potential way to further enhance newsECM labeling and detection sensitivity (Figure S20, Supporting Information). Alternatively, performing newsECM labeling and profiling at different phases of bioengineered tumor tissue culture can provide understanding into the temporal dynamics of tumor cell ECM secretome.

We envision that our newsECM profiling pipeline described herein holds applicability beyond lung cancer. Using MCF‐7, a human breast cancer cell line, we observed robust azido newsECM labeling in both the tumoroid (Figure S21A–C, Supporting Information) and dECM‐tumor (Figure S21D,E, Supporting Information) models administered with Ac_4_GalNAz for 24 h. Beyond synthetic tumor tissues, this presented approach can serve as a platform technology applicable to nearly all cell‐incorporated, tissue‐engineered systems to reveal fundamental mechanisms of cell‐ECM interactions. As demonstrated in the ECM‐free tumoroid model, we also anticipate our approach to facilitate studies in material‐free organoid or spheroid cultures to reveal temporally regulated ECM production in response to induced changes in genetic, environmental, or therapeutic conditions. Building upon our prior study demonstrating the effectiveness of newsECM labeling in a wide variety of tissues and organs in vivo,^[^ ^26^, ^70^ ^]^ we anticipate the chemoselective proteomic pipeline described here to be applicable to animal models of organogenesis and pathogenesis, and deliver mechanistic insights regarding the coordinated cell and ECM remodeling. Lastly, leveraging new developments in ex vivo tissue perfusion and culture (such as precision‐cut tissue slice)^[^ ^71^, ^72^ ^]^ and our recent demonstration of newly synthesized glycoprotein labeling during ex vivo lung perfusion,^[^ ^27^ ^]^ our approach also opens the door to direct inquiry into ECM dynamics in human tissues bearing specific pathophysiological conditions.

## 4 Experimental Section

### Cell Culture

The human bronchioalveolar carcinoma cell line NCI‐H358 (ATCC, CRL‐5807) was grown as adherent cultures in complete medium composed of RPMI‐1640 supplemented with 10% fetal bovine serum (FBS). The human breast adenocarcinoma cell line MCF‐7 (ATCC, HTB‐22) was grown as adherent cultures in complete medium following vendor's suggestions (Eagle's Minimum Essential Medium (EMEM) supplemented with 10% FBS and 1.4 mL Gibco Insulin, human recombinant, zinc solution (4 mg mL^−1^ stock; Thermo Scientific 12585‐014)). All cells used in this study tested negative for mycoplasma. Possible contaminations of stromal cells (fibroblasts, monocytes or macrophages) in NCI‐H358 cell line were excluded by immunofluorescent staining of S100A4 (fibroblast marker) and CD68 (monocyte and macrophage marker) (Figure S22, Supporting Information). For immunofluorescent staining on 2‐D cell culture, the cells in 96‐well plates were washed 3 times with Dulbecco's Phosphate‐Buffered Saline (DPBS) and fixed with 4% paraformaldehyde (PFA) in PBS on ice for 20 min. The fixed cells were subjected to immunofluorescent staining.

### Whole Lung Perfusion Decellularization

All animal experiments were approved by and performed according to the guidelines of the Institutional Animal Care and Use Committee (IACUC) at Carnegie Mellon University under Protocol Number #PROTO201700029. Heart‐lung blocs were isolated from Sprague‐Dawley rats (200‐250g, Charles River Laboratories, Strain Code 400), cannulated and perfusion decellularized as previously described.^[^ ^70^ ^]^ Briefly, rats were euthanized under CO_2_ inhalation, followed by a flushing of PBS through the pulmonary artery (PA) and the isolation of heart‐lung bloc. The PA is then cannulated and perfusion decellularized with 0.1% SDS, followed by sequential washing of water, 1% Triton X‐100 and PBS. The dECM lung scaffolds were preserved in sterile DPBS supplemented with anti‐biotic/anti‐mycotic at 4 °C.

### Establishment of the dECM‐Tumor Model and Metabolic Labeling of Newly Synthesized Proteins

8 dECM lung scaffolds were randomly assigned into 2 groups: 5 for the Ac_4_GalNAz group and 3 for vehicle (DMSO) control group, differentiated by the chemical administration on day 6. The dECM lung scaffolds were perfusion washed with PBS for 30 min before the seeding procedure. 50 million NCI‐H358 cells were collected from culture flasks and resuspended to 50 mL. The dECM scaffolds were flushed with 30 mL DPBS through the trachea under ≈40 cm H_2_O gravity pressure and then seeded with 50 million resuspended cells through the trachea under the same gravity pressure.^[^ ^29^ ^]^ The trachea was ligated after the instillation to maintain airway pressure. The dECM‐tumor was perfusion cultured with complete medium (RPMI‐1640 supplemented with 10% FBS, 100 units mL^−1^ of penicillin and 100 µg mL^−1^ of streptomycin) with the perfusion rate at 1.26 mL min^−1^ in the first 24 h, and 5.04 mL min^−1^ in the following 6 days. A complete medium change was performed every 2 days. On day 6, the medium was changed to complete medium supplemented with Ac_4_GalNAz (Vector Laboratories, CCT‐1086; final concentration 50 µm with 0.1% DMSO) or DMSO (final concentration 0.1%, as vehicle control) and the dECM‐tumors were harvested 24 h later on day 7. For characterization of tumor cell distribution in the dECM scaffold, the dECM‐tumor was perfusion‐cultured for 24 h with complete medium with the perfusion rate at 1.26 mL min^−1^. Upon harvesting, the dECM‐tumors were flushed with 10 mL DPBS through the PA to remove the leftover medium in the scaffold. Then the left lobe and right lower lobe were ligated and removed for protein extraction. The remaining lobes were gravity‐fixed with 4% PFA in PBS for histological analysis. For acellular control of the dECM‐tumor labeling, the dECM lung scaffold was perfusion wash with PBS for 30 min and then subjected to perfusion culture with complete medium supplemented with Ac_4_GalNAz (50 µm with 0.1% DMSO) for 24 h with the perfusion rat at 5.04 mL min^−1^. Upon harvesting, the dECM scaffold was flushed with 10 mL DPBS through the PA to remove the leftover medium and then gravity‐fixed with 4% PFA in PBS for histological analysis. For MCF‐7 dECM‐tumor, the dECM lung scaffold was washed with PBS and seeded with 15 million resuspended MCF‐7 cells through the trachea, followed by trachea ligation. The dECM‐tumor was perfusion cultured with complete medium (EMEM supplemented with 10% FBS and 1.4 mL Gibco Insulin, human recombinant, zinc solution (4 mg mL^−1^ stock)) with the perfusion rate at 1.26 mL min^−1^ in the first 24 h, and 5.04 mL min^−1^ in the following day. The medium was changed to complete medium supplemented with Ac_4_GalNAz (final concentration 50 µm with 0.1% DMSO) on the second day of perfusion culture and administered for 24 h. The MCF‐7 dECM‐tumor was then harvested and flushed with DPBS for subsequent protein extraction.

### Generating NCI‐H358 Tumoroids and Metabolic Labeling of Newly Synthesized Proteins

3.2 million NCI‐H358 cells were resuspended in 4 mL complete medium (RPMI‐1640 supplemented with 10% FBS, 100 units mL^−1^ of penicillin and 100 µg mL^−1^ of streptomycin) into each well of a 6‐well cell‐repellent plate. Every 2 wells of tumoroids were considered as 1 sample for subsequent analysis and 10 samples (from 20 wells) were randomly assigned into 2 groups: 5 samples (from 10 wells) for the Ac_4_GalNAz group and 5 samples (from 10 wells) for vehicle (DMSO) control group, differentiated by the chemical administration on day 6. The plates were placed on an orbital shaker at 125 rpm with half of the medium changed daily for tumoroid formation and maintenance. On day 6, the medium was changed to complete medium supplemented with Ac_4_GalNAz (final concentration 50 µm with 0.1% DMSO) or DMSO (final concentration 0.1%, as vehicle control). After 24 h, the tumoroids were collected and washed with DPBS, followed by protein extraction or fixation under 4% PFA in PBS. The fixed tumoroids were embedded in HistoGel for histological analysis. For medium collection, the medium was refreshed on day 6 and collected after brief centrifugation on day 7 for subsequent western blot analysis. For the competition labeling assay, the tumoroids culture medium was changed to complete medium with 50 µm Ac_4_GalNAz with 0.1% DMSO, 50 µm Ac_4_GalNAz supplemented with 20 mm N‐Acetyl‐D‐galactosamine (GalNAc, Millipore A2795) with 0.1% DMSO, or 0.1% DMSO as vehicle control on day 3. The tumoroids were collected on day 4 and washed for subsequent protein extraction. For extended azido labeling periods, the tumoroids were kept in complete medium supplemented with Ac_4_GalNAz (final concentration 50 µm with 0.1% DMSO) for 48 or 72 h since day 6. During the labeling period, a complete medium change was done every 24 h. After labeling, the tumoroids were collected and washed with DPBS, followed by protein extraction.

### Generating MCF‐7 Tumoroids and Metabolic Labeling of Newly Synthesized Proteins

MCF‐7 cells were lifted and diluted to 0.9 million per mL with complete medium (EMEM supplemented with 10% FBS and 1.4 mL Gibco Insulin, human recombinant, zinc solution (4 mg mL^−1^ stock)). The 24‐well Aggrewell 400 (STEMCELL Technologies, 34411) was coated with Anti‐Adherence Rinsing Solution (STEMCELL Technologies, 07010) and airdried in room temperature. 2 mL of cell suspension (1.8 million) was added to each well (1200 aggregates per well, 1500 cells per aggregate), followed by centrifugation of the Aggrewell plate to capture cells in the microwells for forming tumoroids. The tumoroids were cultured for 3 days with half medium change daily. On day 2, the medium was changed to complete medium supplemented with Ac_4_GalNAz (final concentration 50 µm with 0.1% DMSO) or DMSO (final concentration 0.1%, as vehicle control). After 24 h, the tumoroids were collected and washed with DPBS followed by protein extraction for western blot analysis.

### Histology and Immunostaining

The fixed dECM‐tumors or HistoGel‐embedded tumoroids were sectioned and stained with hematoxylin and eosin (H&E). For immunofluorescence staining on tissue sections, the sections were incubated for antigen unmasking in citrate‐based solution and permeabilized with 0.1% Triton X‐100. For immunofluorescent staining on 2‐D cell culture, the fixed cells were permeabilized with 0.1% Triton X‐100. For immunofluorescence detection of azido signal, the sections were reacted to Alkyne‐PEG4‐Biotin (Vector Laboratories, CCT‐TA105) under copper‐catalyzed cycloaddition (CuAAC) condition.^[^ ^48^ ^]^ The sections were blocked with 1% bovine serum albumin (BSA) in DPBS and stained with streptavidin conjugated to Alexa Fluor‐647 (Thermo Fisher Scientific, S21374) at the dilution of 1:500. For antibody‐based immunofluorescence staining, the sections or cells were blocked with 1% BSA in DPBS and incubated with primary antibody for MMP‐14 (Abcam, ab51074) at the dilution of 1:100, E‐Cad (Cell Signaling Technology, 14472; Abcam, ab40772) at the dilution of 1:200 or 1:100, respectively, hLAMC2 (Abcam, ab210959) at the dilution of 1:500, LAMA1 (Abcam, ab11575) at the dilution of 1:500, or S100A4 (Cell Signaling Technology, 13018) at the dilution of 1:200, CD68 (Thermo Scientific, 14‐0688‐82) at the dilution of 1:100. The sections or cells were stained with secondary antibodies conjugated to Alexa Fluor‐647 or Alexa Fluor‐488 at the dilution of 1:500. All slides were mounted with DAPI‐containing Fluoromount solution (SouthernBiotech, 0100–20). The cells were co‐stained with Hoechst (Thermo Scientific, R37605).

### Immunofluorescence Quantification

Immunofluorescence images were taken under EVOS FL Auto 2 Imaging System (Thermo Fisher Scientific), Nikon A1R HD25 Confocal Microscope System (Nikon Instruments Inc.), or Nikon AX/AX R with NSPARC Confocal Microscope System (Nikon Instruments Inc.). For dECM‐tumor sections, five random fields with the presence of tumor cell clusters (as indicated by DAPI signals) were taken for each biological replicate (the fields within each biological replicate of the dECM‐tumor are indicated in Figure S23, Supporting Information); for tumoroid sections, each tumoroid was considered as a replicate. Fluorescent images were split into their component RGB channels. The blue channel, containing the DAPI signal in all images taken, was converted into a map of the tissue within the image via a custom MATLAB program. The program adjusts the saturation to balance the blue channel before being passed through a customized pixel intensity threshold to filter out noise, binarizing the image in the process. Size filtration was then applied to the map to remove non‐nuclei debris. The identified nuclei blobs were then dilated and eroded by a disk structuring element to connect the blobs in a manner that approximates the underlying tissue, resulting in a “DAPI map”. Finally, the average pixel intensities (normalized to scale of 0 to 1) of the red and green channels (each representing stains for various proteins) both within and outside the approximated tissue area were calculated through logical indexing via this DAPI map. The resulting intensities from red or green channels were then used to calculate normalized immunofluorescence intensities. All immunofluorescence images presented in figures were processed by Fiji software.^[^ ^73^ ^]^

### Stepwise Extraction of Cellular and ECM Protein Fractions and Biotinylation

For the dECM‐tumors, the tissues were minced into fine pieces with surgical scissors and homogenized in 2‐mL tubes prefilled with glass beads (Benchmark Scientific, D1031‐10). The homogenized tissues were washed with DPBS to remove the perfusate residues. The cellular fractions were extracted at 4 °C with 8 mm CHAPS (Thermo Fisher Scientific, 28300) buffer prepared in DPBS (pH 5.50) that is supplemented with 1 m NaCl and 1% protease and phosphatase inhibitor cocktail (Thermo Fisher Scientific, 78440). The insoluble pellets were washed with DPBS, and the ECM fractions were extracted with 4 m Urea (Millipore Sigma, U5128), 0.1% SDS in DPBS supplemented with 1% protease and phosphatase inhibitor cocktail. For the ECM‐free tumoroids, the samples were incubated at 4 °C under agitation with 8 mm CHAPS buffer prepared in DPBS (pH 5.50) supplemented with 1 m NaCl and 1% protease and phosphatase inhibitor cocktail to extract cellular fractions. The insoluble pellets were then washed with DPBS, and the ECM fractions were extracted with 4 m Urea, 0.1% SDS in DPBS supplemented with 1% protease and phosphatase inhibitor cocktail. The extracts were reacted to Alkyne‐PEG4‐biotin under the CuAAC condition for Western blot analysis.

### Western Blots and In‐Gel/Dot Blot Total Protein Staining

Western blotting and in‐gel total protein staining were performed as previously described.^[^ ^27^ ^]^ Briefly, the protein samples were quantified with bicinchoninic acid (BCA) assay, and an equal amount of protein was analyzed using sodium dodecyl‐sulfate polyacrylamide gel electrophoresis (SDS‐PAGE). For Western blot, the proteins in gel were transferred to polyvinylidene difluoride (PVDF) membranes, incubated with primary antibodies for MMP‐14 (Abcam, ab51074) at the dilution of 1:1000, GAPDH (Santa Cruz Biotechnology, sc‐32233) at the dilution of 1:500, LAMA1 (Abcam, ab11575) at the dilution of 1:2000, TUBA1A (Abcam, ab7291) at the dilution of 1:5000, or FN (Abcam, ab2413) at the dilution of 1:500, followed by staining with secondary antibodies conjugated to horseradish peroxidase (HRP) for autoradiography. For in‐gel total protein staining, PAGE gels were fixed with 50% methanol and 7% acetic acid, stained with SYPRO Ruby protein gel stain (Thermo Fisher Scientific, S12000) and visualized under ultraviolet light. For dot blot total protein stain, 3 µL of protein samples were applied to nitrocellulose membranes. The membranes were incubated in 10% methanol, 7% acetic acid, stained with SYPRO Ruby protein blot stain (Thermo Fisher Scientific, S11791) and visualized under ultraviolet light. Image colors of SYPRO ruby stains were inverted. Signal intensities were measured with Fiji software.

### Chemoselective Enrichment of Azido‐Tagged Proteins

The ECM extracts from dECM‐tumors and tumoroids were reacted with Alkyne‐PEG4‐Desthiobiotin (Vector Laboratories, CCT‐1109) under the CuAAC condition. The unreacted Alkyne‐PEG4‐Desthiobiotin was removed with buffer exchange into 5 m Urea, 0.1% SDS in DPBS (pH 7.40) using a 3 kDa molecular weight cut‐off filter (Millipore Sigma, UFC5003), and the retentates were saved as “input” samples. Following quantification by Pierce BCA protein assay kit (Thermo Fisher Scientific, 23225), aliquots of each input sample containing 1 mg of protein (with a final volume of 1.5 mL) were introduced to 50 µL suspending streptavidin‐conjugated resins (Thermo Fisher Scientific, 53116) for pull‐down of desthiobiotinylated proteins. Following 5‐h binding, the supernatants were collected, and the resins were washed 8 times, 15 min per time, with 5 m Urea, 0.1% SDS in DPBS (pH 7.40). The pulled‐down proteins were eluted with 500 µL of 5 m Urea, 0.1% SDS in DPBS (pH 7.40) supplemented with 30 mm biotin at room temperature for 1 h. The resulting eluates were concentrated with a 10 kDa molecular weight cut‐off filter (Millipore Sigma, UFC5010) to 90 µL. 18 µL of each “eluate” sample was used for SDS‐PAGE to test the enrichment efficacy and specificity; the remaining 72 µL was stored at ‐80 °C. For pre‐saturated streptavidin resin control, 10 µL of suspending streptavidin resins were pre‐blocked with 100 µL of 5 m Urea, 0.1% SDS in DPBS (pH 7.40) supplemented with 30 mm biotin at room temperature for 1 h, followed by washing with 5 m Urea, 0.1% SDS in DPBS (pH 7.40) for 5 times, 15 min each. Then tumoroid input samples containing 100 µg of proteins (with a final volume of 300 µL) were introduced to the resins. Following 5‐h binding, the supernatants were collected and subjected to Western blot analysis together with inputs.

### Liquid Chromatography‐Tandem Mass Spectrometry Analysis

The 72 µL “eluate” samples were thawed for proteomic characterization. Analogous “input” samples were prepared by diluting aliquots of each input sample containing ∼3.6 µg of protein (based upon BCA results) to 72 µL with 5 m Urea, 0.1% SDS, in PBS pH 7.40. Disulfide bonds were reduced with 5 mm dithiothreitol (DTT, Millipore Sigma) and heated to 37 °C for 1 h; after cooling, samples were alkylated in the dark for 30 min using 30 mm iodoacetamide (Millipore Sigma). Alkylation was quenched with 5 mm DTT at room temperature for 15 min. Samples were prepared for mass spectrometry using Single‐Pot, Solid‐Phase‐enhanced Sample Preparation (SP3).^[^ ^74^ ^]^ Briefly, samples were reconstituted by adding 100 µg of Sera‐Mag carboxylate‐modified magnetic beads (50 µg/µL, Cytiva) to each sample, followed by an equal volume (90.6 µL) of absolute ethanol (Millipore Sigma). The samples were mixed at room temperature for 5 min on a thermomixer at 1000 rpm to allow protein binding. Afterwards, samples were placed on a magnetic stand for 2 min and the supernatant was removed. Beads were washed three times by sequential resuspension in 180 µL of 80% ethanol, placing on a magnetic stand for 2 min, and removing the supernatant. Proteolytic digestion occurred on the beads after resuspension in 100 µL of 50 mm ammonium bicarbonate (ABC, Millipore Sigma) with 0.14 µg of trypsin (Promega, 1:25 ratio) for the inputs and dECM‐tumor eluates and 0.072 µg of trypsin for the tumoroid eluates. The samples were digested for 2 h at 47 °C, 1000 rpm. Following digestion, the samples were centrifuged at 20,000xg for 1 min at room temperature. The supernatant containing the tryptic peptides was removed, transferred to a new tube, and evaporated to dryness. Samples were reconstituted in 150 µL of 0.1% trifluoracetic acid (TFA) and cleaned up using a C18 ZipTip (Pierce, Thermo Fisher Scientific, 87784) according to the manufacturer's instructions. After evaporation to dryness, samples were reconstituted with 8 µL of 5% acetonitrile containing 0.2% formic acid. Liquid chromatography‐tandem mass spectrometry analysis was performed with 4 µL of each eluate sample and 2 µL of each input sample.

Samples were analyzed by a HPLC‐MS/MS system consisting of a high‐performance liquid chromatograph (HPLC, nanoAcquity, Waters) connected to an electrospray ionization (ESI) Orbitrap mass spectrometer (QE HF, Thermo Fisher Scientific). Injected peptides were loaded onto a 20 cm long fused silica capillary nano‐column packed with C18 beads (1.7 µm in diameter, 130 Angstrom pore size from Waters BEH), with an emitter tip pulled approximately to 1 µm using a laser puller (Sutter instruments). Peptides were initially loaded on‐column for 30 min at 400 nL min^−1^ at 98% buffer A (aqueous 0.1% formic acid) and 2% buffer B (acetonitrile with 0.1% formic acid) and then eluted over 120 min at a flow rate of 300 nL min^−1^ with a gradient as follows: time 1 min‐2% buffer B; time 31 min‐8% buffer B; time 111 min‐44% buffer B; time 122–129 min‐64% buffer B; time 133–152 min‐equilibrate at 2% buffer B. The nano‐column was held at 60 °C using a column heater constructed in house.

The nanospray source voltage was set to 2,200 V. Full‐mass profile scans were performed in the FT‐orbitrap between 375–1,500 m/z at a resolution of 120,000, followed by MS/MS higher‐energy collisional dissociation (HCD) scans of the ten highest intensity parent ions at 30% collision energy (CE) and 15,000 resolution, with a mass range starting at 100 m/z and a 2.5 m/z isolation window. Charge states 2–5 were included and dynamic exclusion was enabled with a repeat count of one over a duration of 15 s. The MS/MS orbitrap HCD scans were collected with automatic gain control (AGC) target set to 1e5 ions, a maximum inject time of 500 ms, and 1 microscan per spectrum.

### Proteomic Data Analysis

LC‐MS/MS data were analyzed with the open search software program MetaMorpheus (version 1.0.5, available at https://github.com/smith‐chem‐wisc/MetaMorpheus). The Swiss‐Prot Rat XML (reviewed) database containing 8,207 protein entries [downloaded from UniProt (UniProtKB, 10116) 04/01/2024] and the Swiss‐Prot Human XML (reviewed) database containing 20,435 protein entries [downloaded from UniProt (UniProtKB, 9606) 04/01/2024] were utilized along with the MetaMorpheus default contaminants database. The UniProt XML databases include not only protein sequences, but also curated and well‐annotated information on observations of post‐translational modifications (PTMs) on specific amino acid residues, such as phosphorylations on serine/threonine/tyrosine and hydroxylations on prolines/lysines. MetaMorpheus was used to calibrate the raw data files for the 36 samples (18 inputs and 18 eluates). Calibrated files were then subjected to global posttranslational modification discovery (GPTMD)^[^ ^75^ ^]^ which searches the mass spectral files for the presence or absence of the PTMs annotated on specific residues per the XML file.^[^ ^76^ ^]^ GPTMD was also used to search and identify possible PTMs not already annotated in the employed databases.^[^ ^75^ ^]^ The modifications searched for in GPTMD included: the Common Biological, Common Artifact, and Metal Adduct categories within MetaMorpheus, as well as custom modifications for the azido‐glycan labels (on serine/threonine with and without the alkyne‐PEG4‐desthiobiotin) at +244.0808 Da and +671.3490 Da (see Table S3, Supporting Information for a detailed listing). The files were then searched against the GPTMD XML databases using the following search parameters: protease = trypsin; search for truncated proteins and proteolysis products = False; maximum missed cleavages = 2; minimum peptide length = 7; maximum peptide length = unspecified; initiator methionine behavior = Variable; fixed modifications = Carbamidomethyl on C, Carbamidomethyl on U; variable modifications = Oxidation on M; max mods per peptide = 2; max modification isoforms = 1024; precursor mass tolerance = ±5.0000 ppm; product mass tolerance = ±20.0000 ppm; report PSM ambiguity = True.

Using the above parameters, the following three searches were completed in MetaMorpheus with label‐free quantification (LFQ) enabled: (1) all 18 eluate files underwent GPTMD and were searched using the Human XML database with Match‐Between‐Runs (MBR), (2) all 16 dECM‐tumor files (inputs and eluates) underwent GPTMD analysis and were searched using the Human and Rat XML databases without MBR, and (3) the 18 dECM‐tumor and tumoroid input files underwent GPTMD analysis and were searched using the Human and Rat XML databases with MBR. Mass spectrometry data has been deposited to the ProteomeXchange Consortium via the PRIDE repository with the data set identifier PXD061871.

Data analysis details for *Search 1* (all 18 eluates) are as follows. All 18 eluates files underwent GPTMD and were searched using the Human XML database with MBR. A one‐way ANOVA was done on protein IDs from each sample across 4 treatment groups (Ac_4_GalNAz, dECM‐tumor; Ac_4_GalNAz, tumoroid; vehicle, dECM‐tumor; vehicle, tumoroid) and outliers were removed for subsequent analyses. The intensities of all human proteins were summed for each sample. For comparison between Ac_4_GalNAz and vehicle groups, the protein intensities were normalized separately in each treatment group. The normalized intensity (Table S4, Supporting Information – “*Search 1* normalization 1”) of protein *X* in sample *i* under treatment group *K* (*K* can be one of the following: Ac_4_GalNAz, dECM‐tumor; Ac_4_GalNAz, tumoroid; vehicle, dECM‐tumor; vehicle, tumoroid) was calculated with the following formula:

$\;X_{Ki,\;normalized}=X_{Ki,original}\;\times \frac{Sum_{average}}{Sum_{Ki}}$, where *Sum_average_* was the average sum of all eluate samples in treatment group *K*.

The dataset was then separated into two subsets based on their culture models (dECM‐tumor or tumoroid) for subsequent analysis. Using Perseus^[^ ^77^ ^]^ each subset was pre‐filtered to remove proteins with less than two nonzero values in each treatment group. Analysis in Perseus continued with the filtered list of protein intensities by first performing a Log_2_ transform and then undergoing a two‐tailed Welch's *t*‐test using criteria in the Statistical Analysis section below.

Another comparison from the *Search 1* dataset was between Ac_4_GalNAz dECM‐tumor and Ac_4_GalNAz tumoroid groups. Proteins from these 9 samples were normalized all together for their proportional intensities in each group. The normalized intensity (Table S4, Supporting Information – “*Search 1* normalization 2”) of protein *X* in sample *j* was calculated with the following formula:

$\;X_{j,\;normalized}=X_{j,original}\;\times \frac{Sum_{average}}{Sum_{j}}$, where *Sum_average_* was the average sum of all (both dECM‐tumor and tumoroid) Ac_4_GalNAz eluate samples. The normalized intensities were used for Gene Ontology analysis. For volcano plots and Venn diagrams, the normalized protein intensities were taken as Log_2_ and imputed using the LMCD‐QRILC R script plugin in Perseus.^[^ ^78^, ^79^ ^]^ Briefly, the impute QRILC script imputed missing values by randomly drawing from a truncated normal distribution, that was estimated using quantile regression. Following imputation, the data underwent two‐tailed Welch's *t*‐test using criteria in the Statistical Analysis section below. For individual protein intensity bar graphs, the imputed intensity values were transformed back to decimal numbers for plotting.

Data analysis details for *Search 2*: One eluate sample was removed as outlier, and the other 15 dECM‐tumor files (inputs and eluates) underwent GPTMD analysis and were searched using the Human and Rat XML databases without MBR: Data was analyzed as original value (Table S4, Supporting Information – “*Search 2* original”) for calculations of total *Homo sapiens* and *Rattus Norvegicus* protein intensities.

Data analysis details for *Search 3*: All 18 tumoroid and dECM‐tumor input files underwent GPTMD analysis and were searched using the Human and Rat XML databases with MBR: The intensities of all human and rat proteins were summed for each sample. All input samples were normalized together. The normalized intensity (Table S4, Supporting Information – “*Search 3* normalization”) of protein *X* in sample *m* was calculated with the following formula:

$\;X_{m,\;normalized}=X_{m,original}\;\times \frac{Sum_{average}}{Sum_{m}}$, where *Sum_average_* was the average sum of all input samples. The normalized intensities were used for Gene Ontology analysis.

### Protein Composition and Kyoto Encyclopedia of Genes and Genomes (KEGG) Pathway Analysis

The KEGG pathway and protein composition analysis was performed and visualized on the Proteomaps website (https://www.proteomaps.net/index.html).^[^ ^33^ ^]^ The protein list along with their normalized values were uploaded and analyzed for “*H. Sapiens*”.

### Gene Ontology Analysis

Gene ontology (GO) analysis was performed on the Database for Annotation, Visualization, and Integrated Discovery Bioinformatics Resources (https://david.ncifcrf.gov/tools.jsp).^[^ ^35^, ^36^ ^]^ The Uniprot ID lists were uploaded and analyzed for functional annotation clustering or enriched for biological process terms. The top annotation clusters with highest enrichment scores were presented in a table. The top GOTERM_BP_DIRECT or GOTERM_CC_DIRECT terms with lowest *p* values were plotted in bar graphs.

### Extracellular Focused Analysis of Proteomics Data

The GO annotations of reviewed human proteins were downloaded from Swiss‐prot containing 20421 protein entries [downloaded from UniProt 05/27/2025]. The list of proteins localizing at the extracellular space were filtered by GO annotations containing “extracellular”. The newsECM proteomes from dECM‐tumors and tumoroids were then filtered for extracellular proteins and subjected to extracellular focused GO analysis.

### Protein‐Protein Interaction Analysis

Protein‐protein interaction analysis was performed with consultation from MatrixDB database^[^ ^80^ ^]^ (https://matrixdb.univ‐lyon1.fr/). Protein accession number was set as “P50281: MMP14_HUMAN”. 38 proteins with experimentally supported interactions and 280 proteins with predicted interactions with MMP‐14 were downloaded. The MI scores (quantitative estimates of the confidence in each interaction) were determined for experimentally supported interactions only.

### Statistical Analysis

The detailed method for proteomics data processing (including database searching, normalization, and imputation) can be found in “Proteomic Data Analysis” section. For all experiments, the *n* values stated represent the number of independent biological samples. Data was analyzed using GraphPad Prism, Microsoft Excel, RStudio, MATLAB, or Perseus. Statistical comparisons of data with three or more treatment groups were performed with one‐way ANOVA with Tukey's multiple comparisons tests (confidence level: 0.95). Between‐group correlation coefficients were analyzed by Pearson correlation test. Outliers were identified and cleared out by Grubbs's tests (*α* = 0.05). The multiple comparison testing of protein intensities for volcano plots and Venn diagrams were analyzed by two‐tailed *t*‐tests with Welch's correction, followed by multiple hypothesis testing correction using a permutation‐based false discovery rate (FDR) calculation. *q*‐values were reported after permutation‐based FDR correction; proteins with *q* < 0.05 (i.e., 5% FDR) were considered significant. For volcano plots, the Perseus output of ‐Log_10_(Welch's *t*‐test *p*‐value) was plotted versus the fold changes (FCs) calculated from geometric means of the decimal protein intensities. Paired measurements were analyzed by paired *t*‐tests. All other comparisons with two treatment groups were analyzed by two‐tailed *t*‐tests with Welch's correction. Error bars were plotted by standard deviations (SD). All statistical significances were reported accordingly. ns, not statistically significant, *p* > 0.05; *p < 0.05; **p < 0.01; ***p < 0.001; ^#^ *q* < 0.05.^[^ ^81^ ^]^

### Schematics

All schematics were created with BioRender.com.

## Conflict of Interest

The authors declare no conflict of interest.

## Supporting information



Supporting Information

## Data Availability

The data that support the findings of this study are available from the corresponding author upon reasonable request. Mass spectrometry data has been deposited to the ProteomeXchange Consortium via the PRIDE partner repository with the data set identifier PXD061871.
