# Supplementary material for: Chemoselective Characterization of New Extracellular Matrix Deposition in Bioengineered Tumor Tissues
Source: Adv Mater. 2025 Sep 6;37(47):e05445. doi: 10.1002/adma.202505445 (PMC12500236; doi:10.1002/adma.202505445)
Supplement: Supplementary file 1 — Supporting Information [file ADMA-37-e05445-s001.docx]

**Chemoselective Characterization of New Extracellular Matrix Deposition in Bioengineered Tumor Tissues**

*Zihan Ling, Burke Niego, Qingyang Li, Dhruv Bhattaram, Vanessa Serna Villa, Michael Hu, Zhuowei Gong, Lloyd M. Smith, Brian L. Frey^*^, and Xi Ren^*^*

Z.L., Q.L., V.S.V., D.B., M.H., Z.G., X.R.

Department of Biomedical Engineering, Carnegie Mellon University, Pittsburgh, Pennsylvania, United States

B.N., L.M.S., B.L.F.

Department of Chemistry, University of Wisconsin, Madison, Wisconsin, United States

^*^ Correspondence: Xi Ren ([xiren@cmu.edu](mailto:xiren@cmu.edu)); Brian L. Frey ([bfrey@chem.wisc.edu](mailto:bfrey@chem.wisc.edu)).

Z.L. and B.N. contributed equally to this work.

**Supporting Information**

**Supplementary figure 1.** Hematoxylin and eosin (H&E) staining of the dECM-tumor at 1 day post cell delivery.

**Supplementary figure 2.** Immunofluorescence staining of azido→biotin (red) and LAMA1 (green) on dECM-tumors or dECM lung scaffolds receiving Ac_4_GalNAz administration during 24-hour perfusion culture.

**Supplementary figure 3.** Western blot detection of Tubulin alpha-1a chain (TUBA1A) and Fibronectin (FN) signal in cellular and ECM fractions of tumoroids.

**Supplementary figure 4.** Western blot detection of azido→biotin signal in cellular fractions of dECM-tumors.

**Supplementary figure 5.** Western blot detection of azido→biotin signal in cellular fractions of tumoroids.

**Supplementary figure 6.** Competition assay for Ac_4_GalNAz labeling. Tumoroids were administered with Ac_4_GalNAz with (*n*=3) or without (*n*=3) GalNAc competition, or administered with DMSO as vehicle control (*n*=3) for 24 hours.

**Supplementary figure 7.** Streptavidin resins pre-blocked with biotin were unable to pull down azido-tagged, desthiobiotinylated newsECM from tumoroids receiving Ac_4_GalNAz administration.

**Supplementary figure 8.** Bar graph of human or rat protein intensities in eluate versus input from the dECM-tumors (*n*=4).

**Supplementary figure 9.** An individual-protein-intensity Proteomap generated with all eluate proteins from the dECM-tumors receiving Ac_4_GalNAz.

**Supplementary figure 10.** Scatter plots of normalized protein intensities in eluate samples from dECM-tumors or tumoroids.

**Supplementary figure 11.** GO analysis of top enriched cellular component terms of the 100 proteins with highest intensities in eluate samples from dECM-tumors (A) or tumoroids (B) receiving Ac_4_GalNAz.

**Supplementary figure 12.** GO analysis of top enriched biological process terms of the 100 extracellular proteins with highest intensities in eluates samples from dECM-tumors (A) or tumoroids (B) receiving Ac_4_GalNAz.

**Supplementary figure 13.** Confocal imaging of immunofluorescence staining of MMP-14 (red) and E-Cad (green) on dECM-tumors.

**Supplementary figure 14.** Western blot detection and quantification of MMP-14 signals in the ECM fractions from dECM-tumors (*n*=3) or tumoroids (*n*=3).

**Supplementary figure 15.** Western blot detection and quantification of MMP-14 signals in dECM-tumor ECM (*n*=3), tumoroid ECM (*n*=3), and tumoroid medium (*n*=3) on day 7 of culture (A).

**Supplementary figure 16.** Immunofluorescence staining of hLAMC2 (red) and E-Cad (green) on dECM-tumors.

**Supplementary figure 17.** Bar graphs showing the normalized, imputed protein intensities of PROX-1 in eluate samples from dECM-tumors (left, red, *n*=4) and tumoroids (right, blue, *n*=5).

**Supplementary figure 18.** Protein-protein physical interaction network functional enrichment analysis with the MatrixDB database.

**Supplementary figure 19.** GO analysis of top enriched cellular component terms of the 100 proteins with highest intensities in input samples from dECM-tumors (A) or tumoroids (B).

**Supplementary figure 20.** Western blot detection of azido→biotin signal in ECM fractions of tumoroids administered with Ac_4_GalNAz for 24, 48 or 72 hours.

**Supplementary figure 21.** Western blot detection of azido→biotin signal in ECM fractions of tumoroids or dECM-tumors, derived from MCF-7 cells, administered with Ac_4_GalNAz.

**Supplementary figure 22.** Immunofluorescence staining of E-Cad (epithelial marker, red), S100A4 (fibroblast marker, red), or CD68 (monocyte and macrophage marker, red) on NCI-H358 cells.

**Supplementary figure 23.** Stitched images of immunofluorescence staining of azido→biotin (red) and LAMA1 (green) on dECM-tumors receiving Ac_4_GalNAz (n=3) or DMSO (vehicle control, n=3) during the last day of culture.

**Table 1.** Intracellular marker intensities in proportion to total human protein intensities identified in dECM-tumor or tumoroid inputs.

**Table 2.** Functional annotation clustering of proteins with top 100 abundance from dECM-tumor newsECM.

**Table 3.** MetaMorpheus modifications for G-PTM-D.

**Table 4.** Individual protein intensities before or/and after normalization in all three searches.


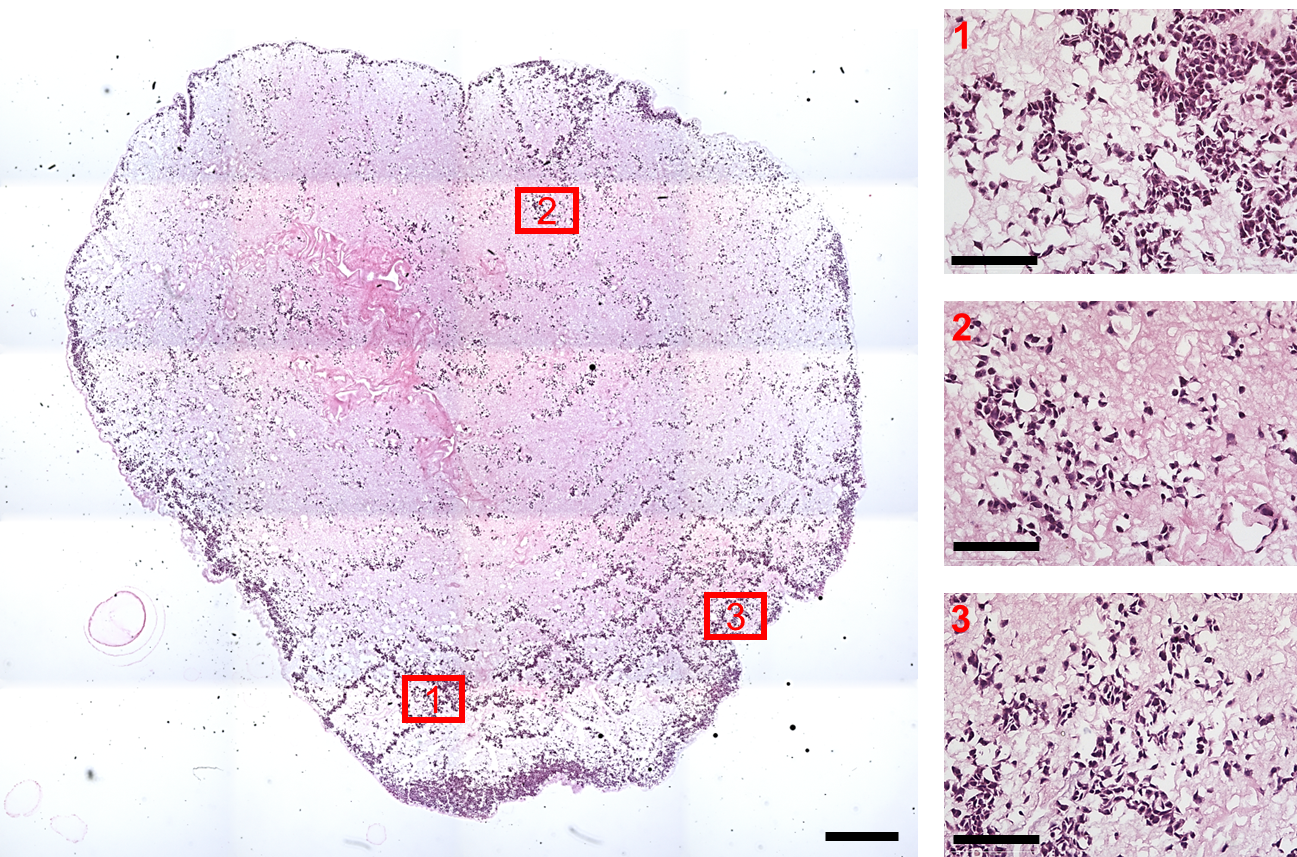


**Supplementary figure 1. Hematoxylin and eosin (H&E) staining of the dECM-tumor at 1 day post cell delivery.** Random regions in the whole-lobe scan (left) were selected for close-up views (right). Scale bars: left, 1000 µm; right, 150 µm.


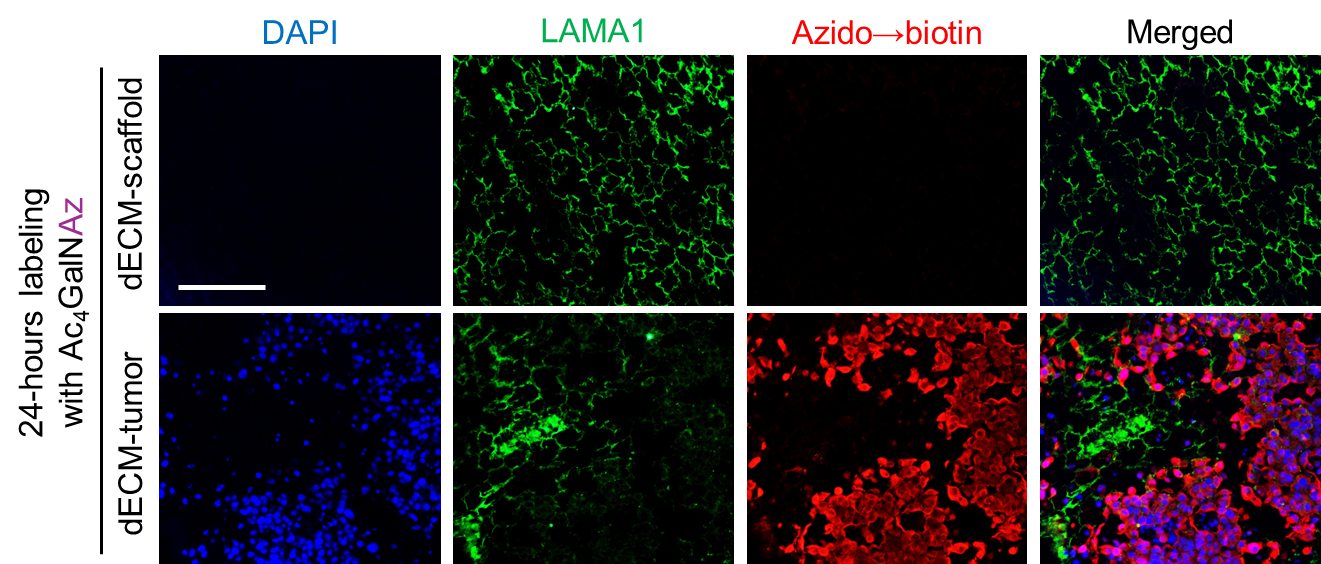


**Supplementary figure 2.** **Immunofluorescence staining of azido→biotin (red) and LAMA1 (green) on dECM-tumors or dECM lung scaffolds receiving Ac_4_GalNAz administration during 24-hour perfusion culture.** Scale bar, 150 µm.


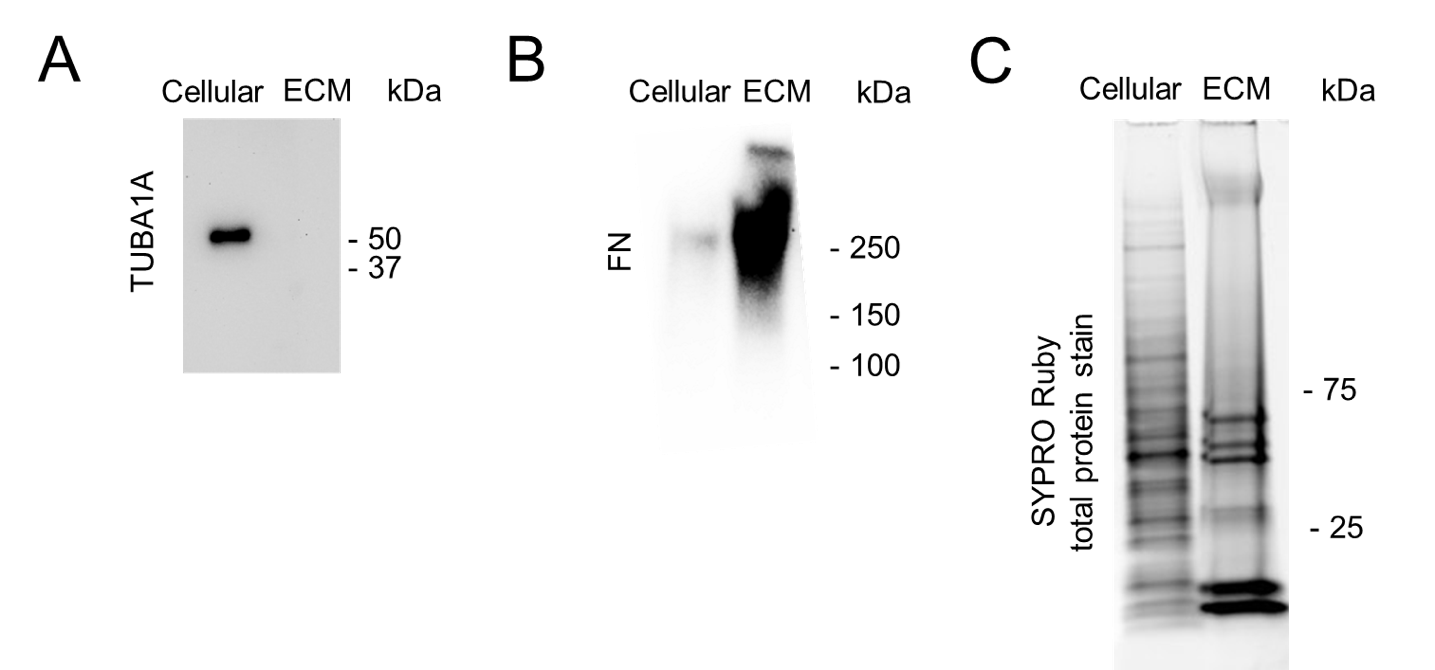


**Supplementary figure 3.** **Western blot detection of Tubulin alpha-1a chain (TUBA1A, A) and Fibronectin (FN, B) signal in cellular and ECM fractions of tumoroids.** (C) SYPRO Ruby staining of total proteins.


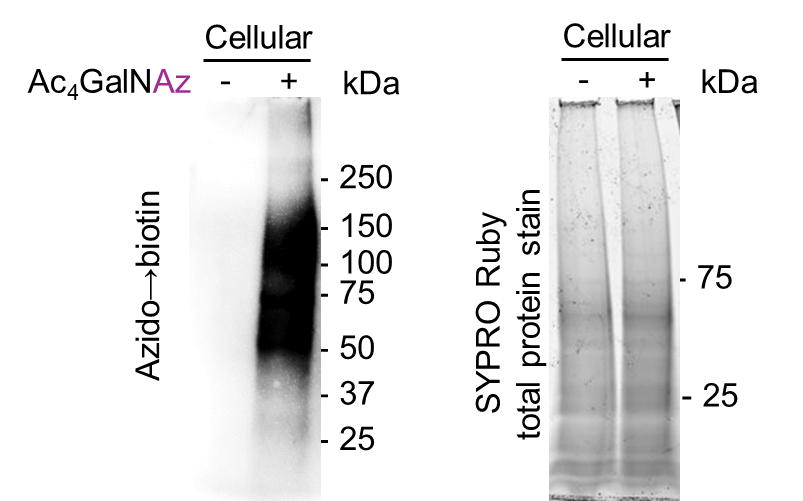


**Supplementary figure 4.** **Western blot detection of azido→biotin signal in cellular fractions of dECM-tumors.** Azido→biotin signal detected using streptavidin-HRP (left) and SYPRO Ruby staining of total proteins (right).


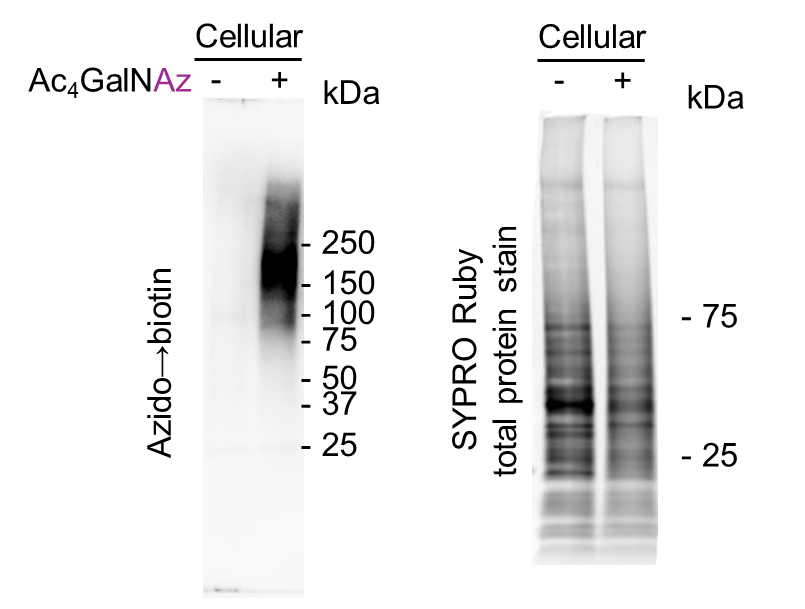
**Supplementary figure 5.** **Western blot detection of azido→biotin signal in cellular fractions of tumoroids.** Azido→biotin signal detected using streptavidin-HRP (left) and SYPRO Ruby staining of total proteins (right).


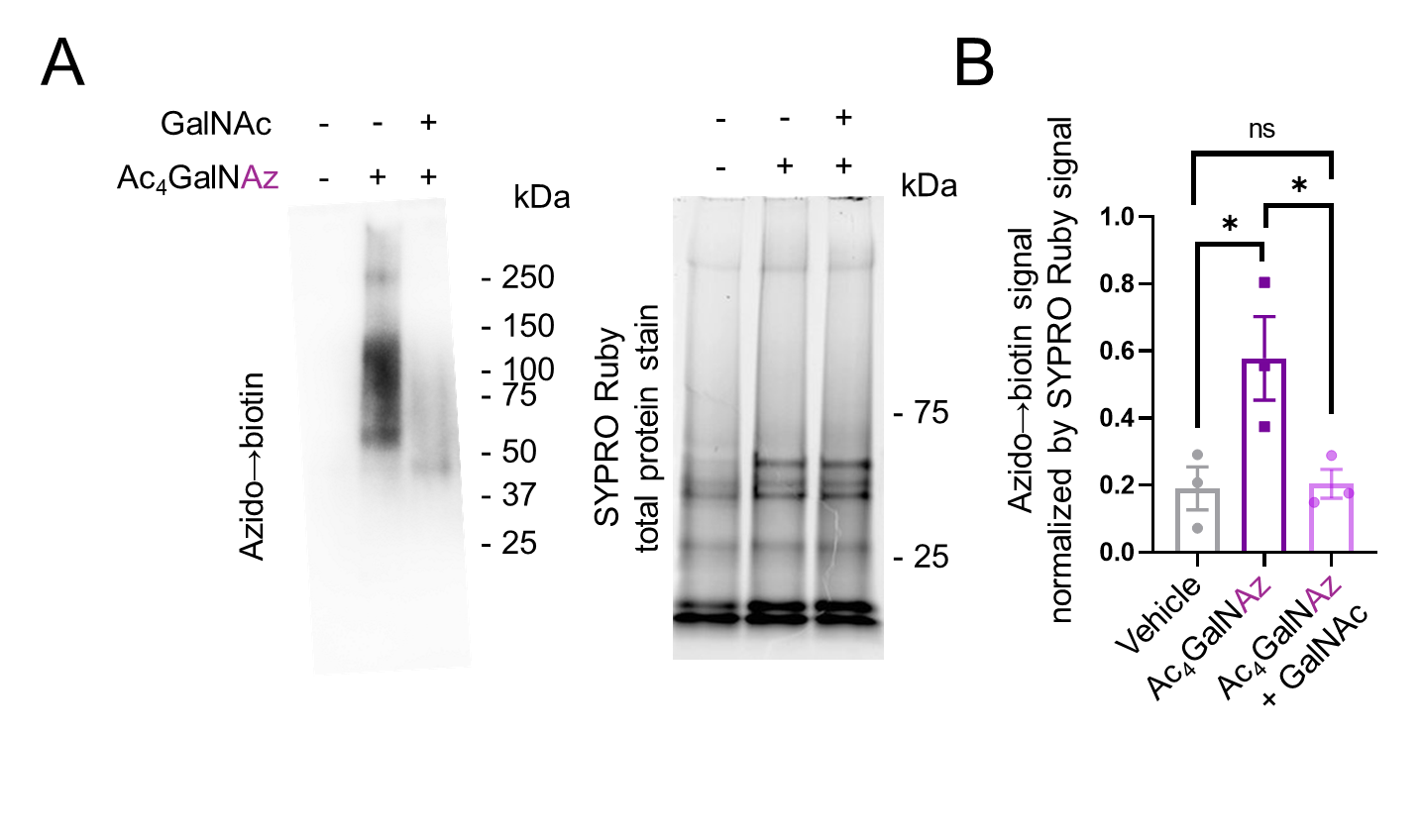


**Supplementary figure 6.** **Competition assay for Ac_4_GalNAz labeling. Tumoroids were administered with** **Ac_4_GalNAz with (*n*=3) or without (*n*=3) GalNAc competition, or administered with DMSO as vehicle control (*n*=3) for 24 hours.** (A) Western blot analysis of biotin signal (left) and SYPRO Ruby staining of total proteins (right) in tumoroid ECM fractions administered with different probe conditions. (B) Quantification of azido→biotin signals in panel A (left), normalized by total protein intensities in panel A (right). The data was analyzed by one-way ANOVA with Tukey’s multiple comparisons tests (confidence level: 0.95). ns, *p* > 0.05; **p* < 0.05. Data are presented as means ± SD.


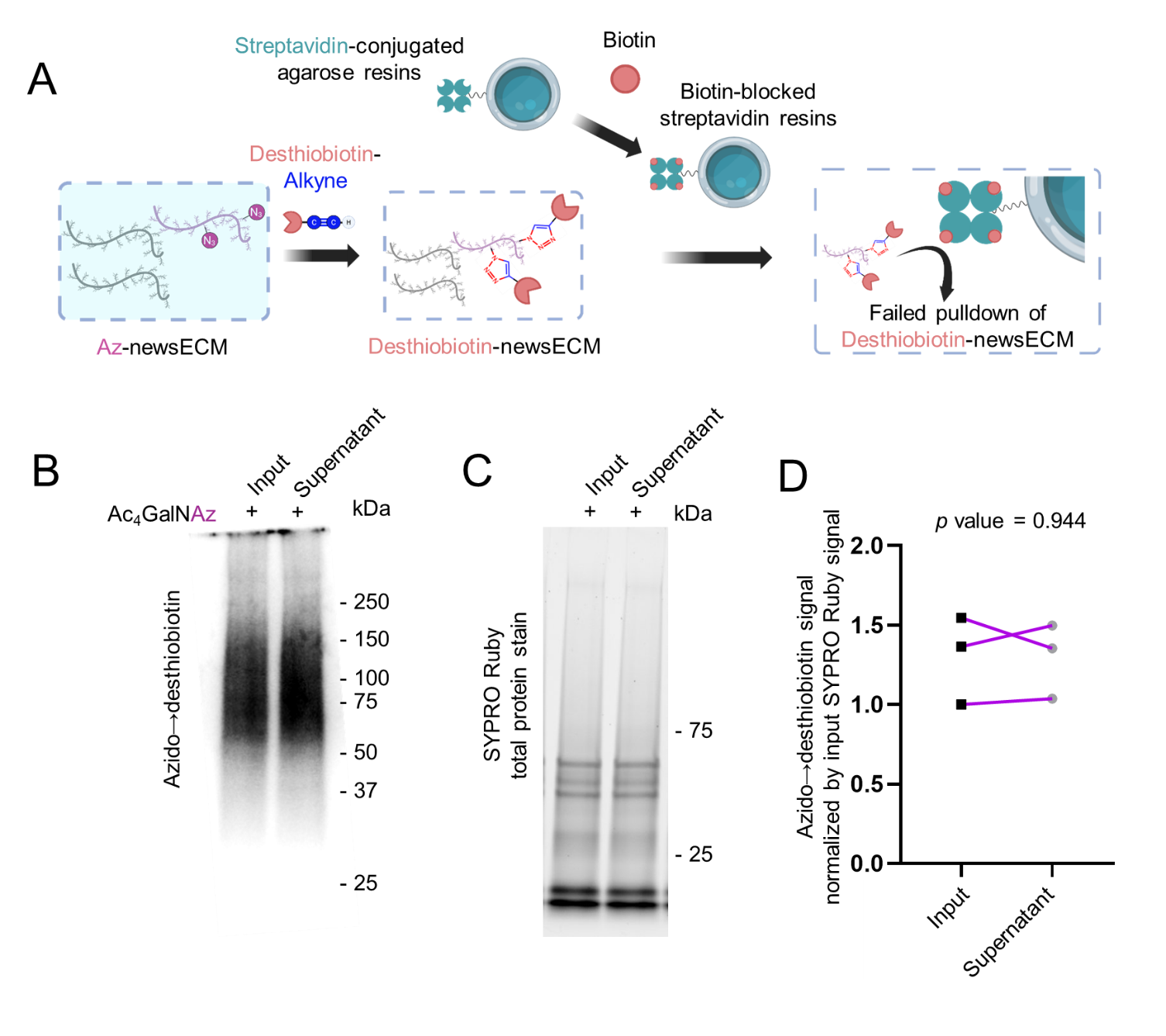


**Supplementary figure 7.** **Streptavidin resins pre-blocked with biotin were unable to pull down azido-tagged, desthiobiotinylated newsECM from tumoroids receiving Ac_4_GalNAz administration.** (A) A Schematic showing the workflow for tumoroid newsECM binding with streptavidin resins pre-blocked with free biotin, (B-C) Western blot analysis of desthiobiotin signal (B) and SYPRO Ruby staining of total proteins (C) in tumoroid ECM fractions before (input, left, *n*=3) or after (supernatant, right, *n*=3) incubation with streptavidin resins pre-treated with free biotin. (D) Quantification of desthiobiotin intensities in panel B, normalized by input total protein intensities in panel C. The data was analyzed by paired *t*-test. Schematics were created with Biorender.com and published with permission.


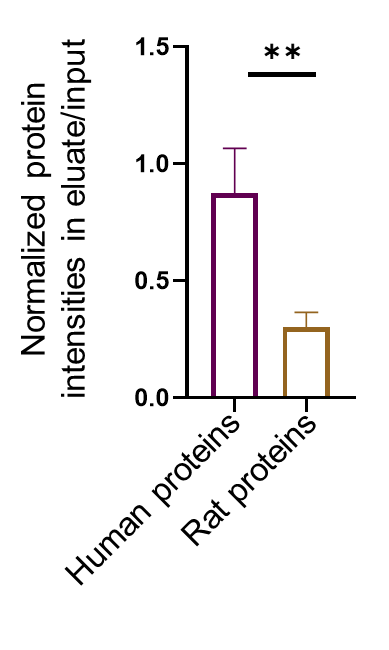
**Supplementary figure 8.** **Bar graph of human or rat protein intensities in eluate versus input from the dECM-tumors (*n*=4).** The data was analyzed by two-tailed *t*-tests with Welch’s correction. ** *p*<0.01. Data are presented as means ± SD.


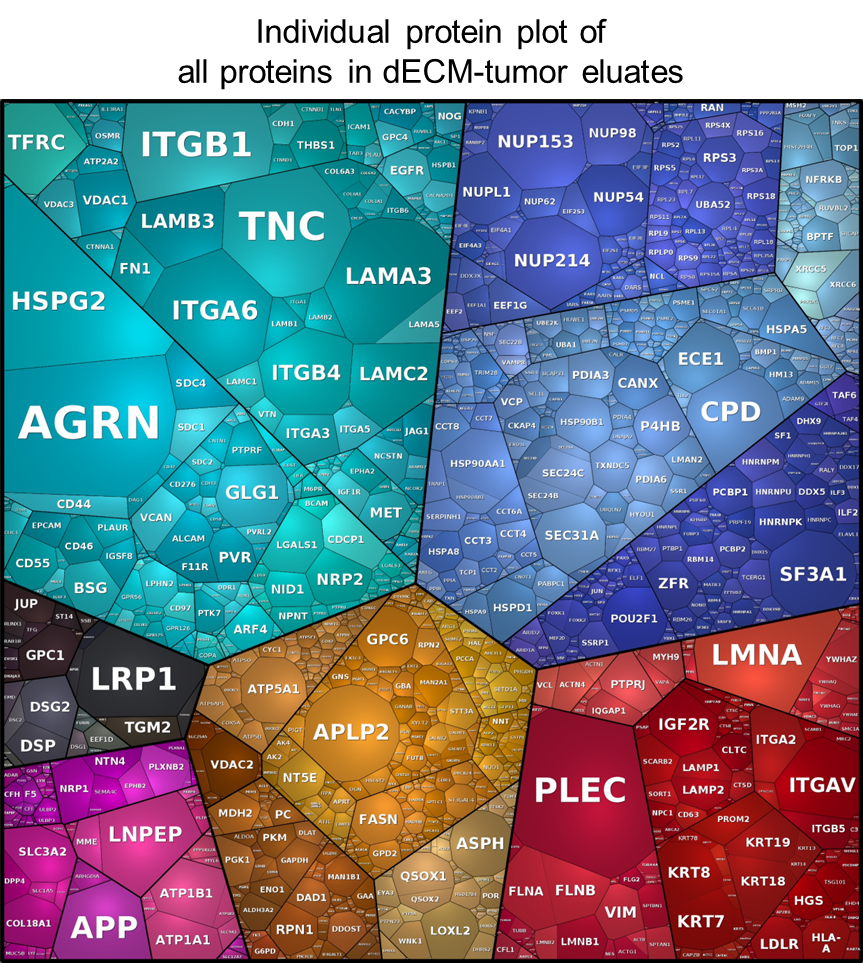


**Supplementary figure 9.** **An individual-protein-intensity Proteomap generated with all eluate proteins from the dECM-tumors receiving Ac_4_GalNAz.** The area of each protein represents its intensity level and color-coded for different proteins.

**
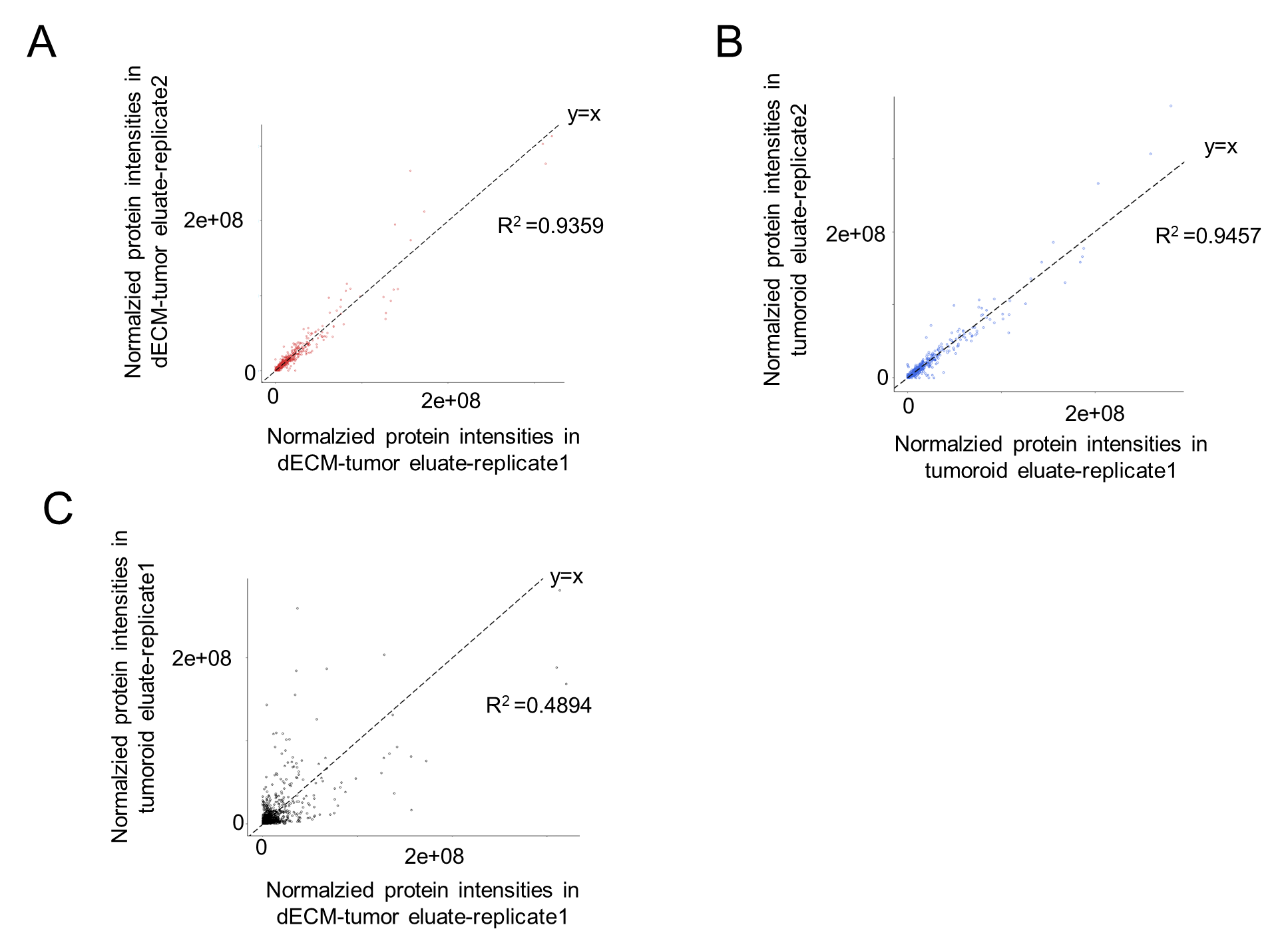
Supplementary figure 10. Scatter plots of normalized protein intensities in eluate samples from dECM-tumors or tumoroids.** Scatter plots of protein intensities between (A) two eluate samples from dECM-tumors, (B) two eluate samples from tumoroids, and (C) one eluate sample from dECM-tumors and one eluate sample from tumoroids.


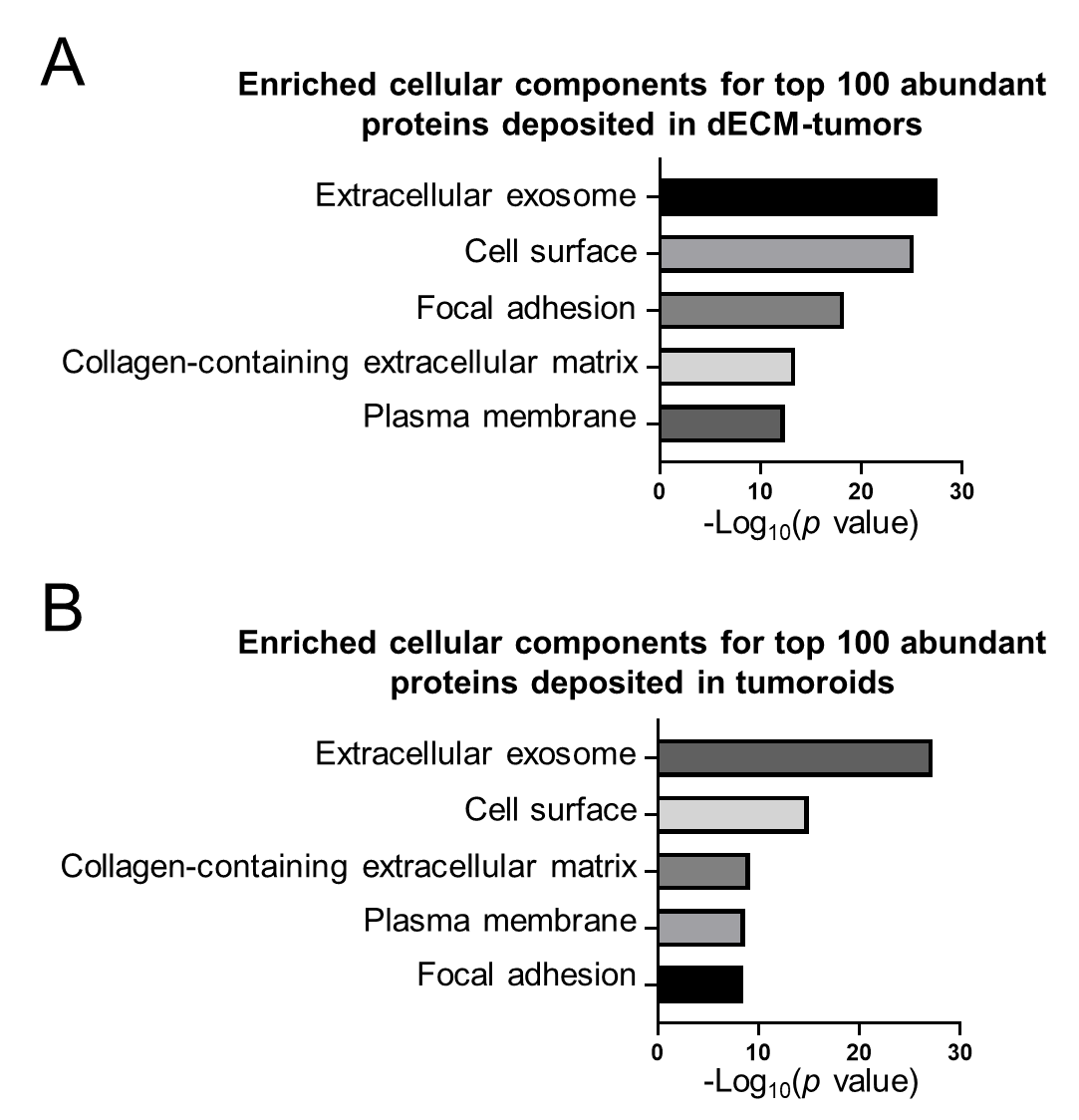


**Supplementary figure 11. GO analysis of top enriched cellular component terms of the 100 proteins with highest intensities in eluates samples from dECM-tumors (A) or tumoroids (B) receiving Ac_4_GalNAz.**


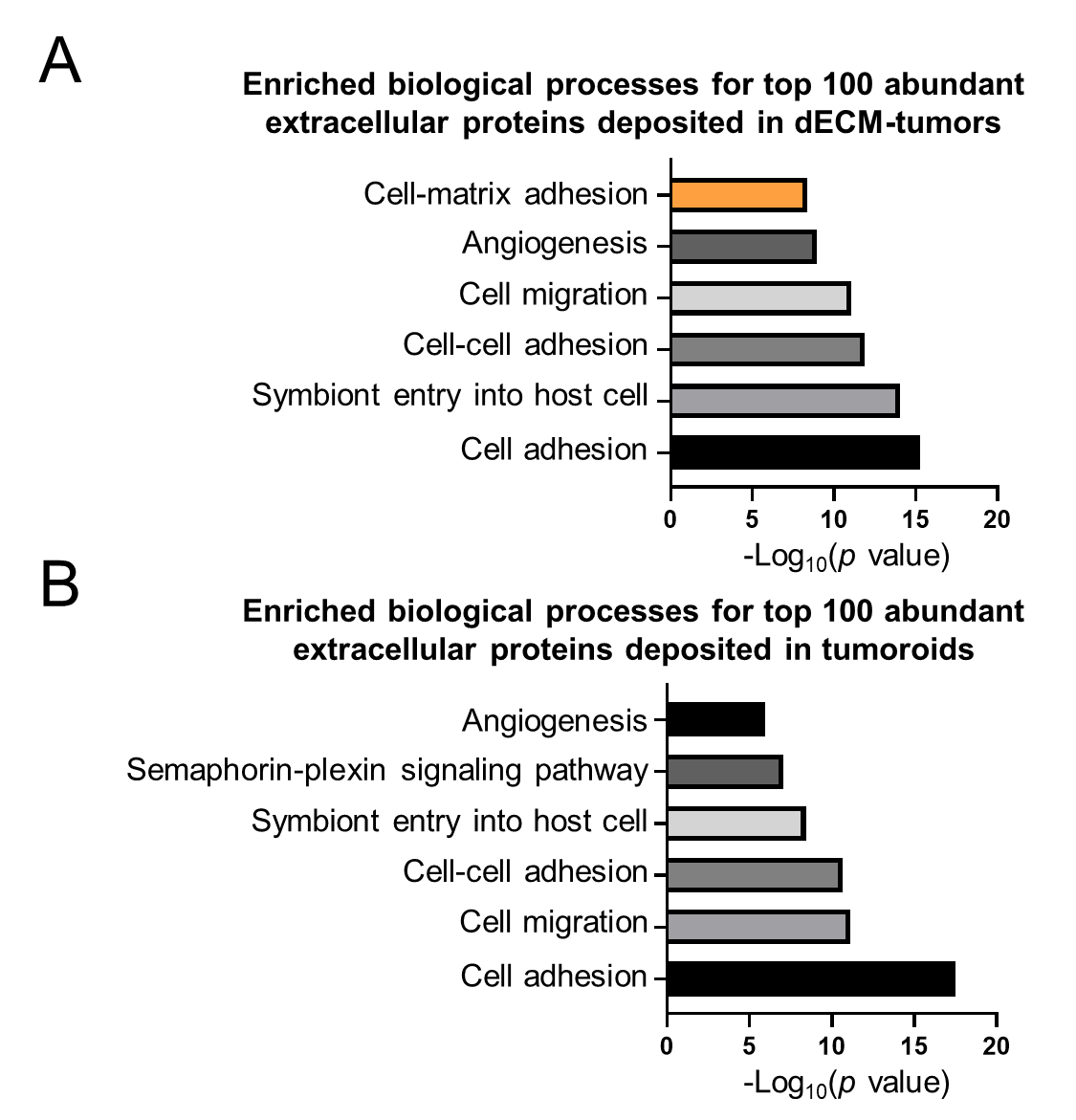


**Supplementary figure 12. GO analysis of top enriched biological process terms of the 100 extracellular proteins with highest intensities in eluates samples from dECM-tumors (A) or tumoroids (B) receiving Ac_4_GalNAz.**


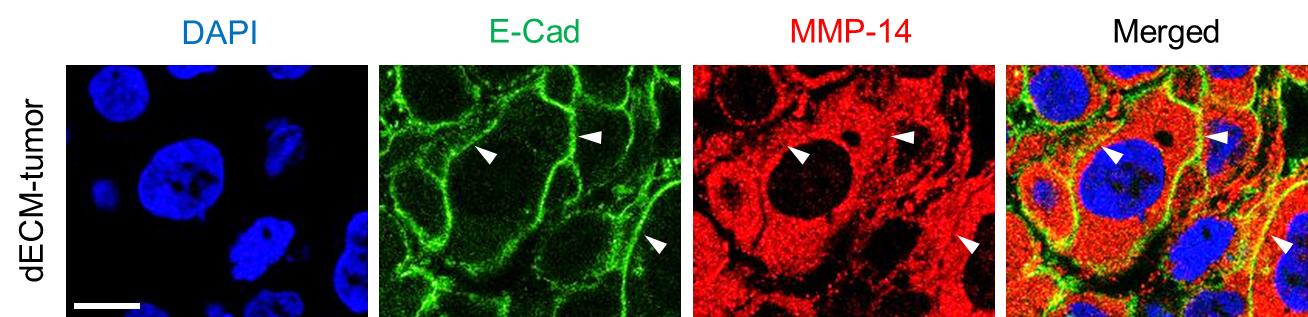


**Supplementary figure 13.** **Confocal imaging of immunofluorescence staining of MMP-14 (red) and E-Cad (green) on dECM-tumors.** Arrowheads indicate MMP-14 signals with E-Cad co-localization on the plasma membrane. Scale bar, 25 µm.

**
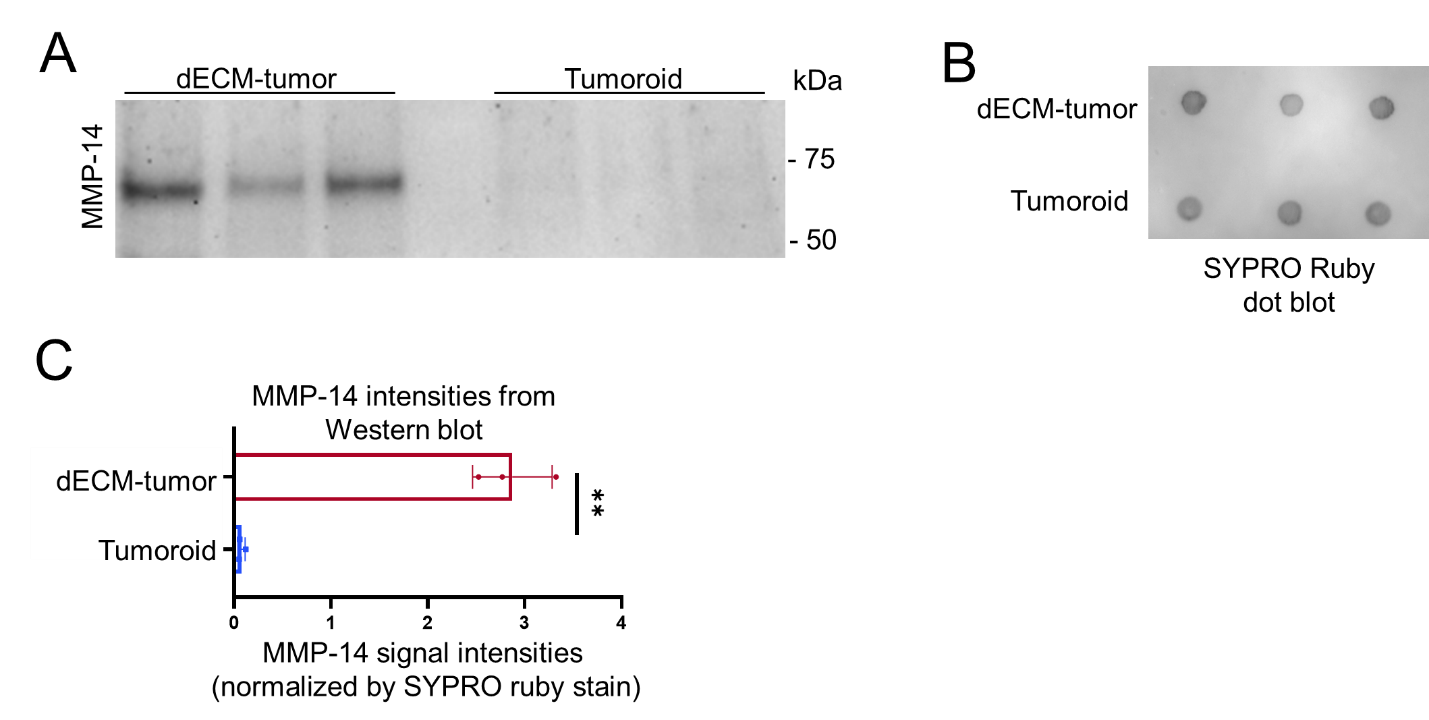
**

**Supplementary figure 14. Western blot detection and quantification of MMP-14 signals in the ECM fractions from dECM-tumors (*n*=3) or tumoroids (*n*=3).** (A) Western blot detection of MMP-14 in the ECM fractions from dECM-tumors (left) or tumoroids (right). (B) SYPRO Ruby dot blot of total proteins from each sample analyzed in panel A. (C) Quantification of MMP-14 signal intensities in panel A, normalized by SYPRO Ruby dot blot signals in panel B. The data was analyzed by two tailed *t* tests with Welch’s correction. ** *p*<0.01. Data are presented as means ± SD.


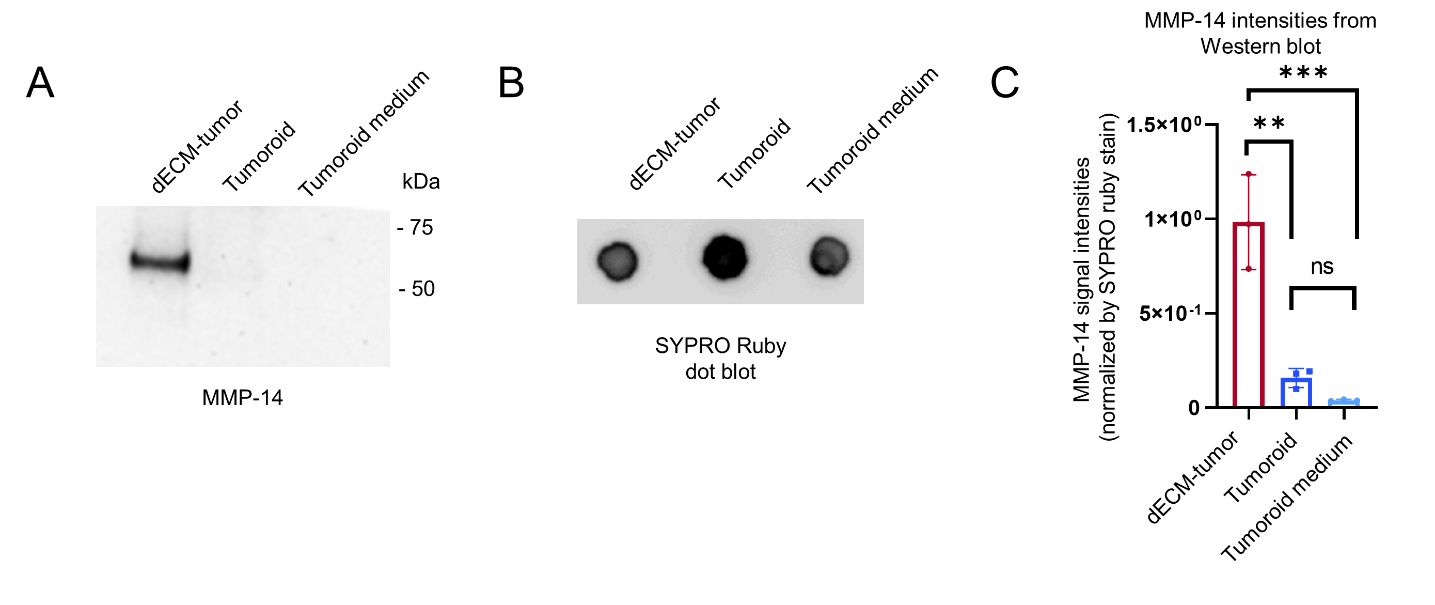


**Supplementary figure 15. Western blot detection and quantification of MMP-14 signals in dECM-tumor ECM (*n*=3), tumoroid ECM (*n*=3), and tumoroid medium (*n*=3) on day 7 of culture (A).** (B) SYPRO Ruby dot blot analysis of total proteins from each sample analyzed in panel A. (C) Quantification of MMP-14 signal intensities in panel A, normalized by SYPRO Ruby dot blot signals in panel B. The data was analyzed with one-way ANOVA with Tukey’s multiple comparisons tests (confidence level: 0.95). *** *p*<0.001; ** *p*<0.01; ns, not statistically significant, *p*>0.05. Data are presented as means ± SD.


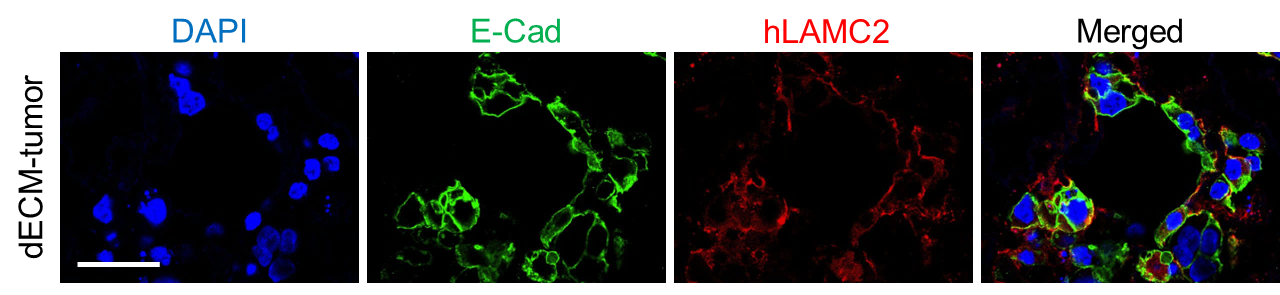


**Supplementary figure 16.** **Immunofluorescence staining of hLAMC2 (red) and E-Cad (green) on dECM-tumors.** Scale bar, 50 µm.


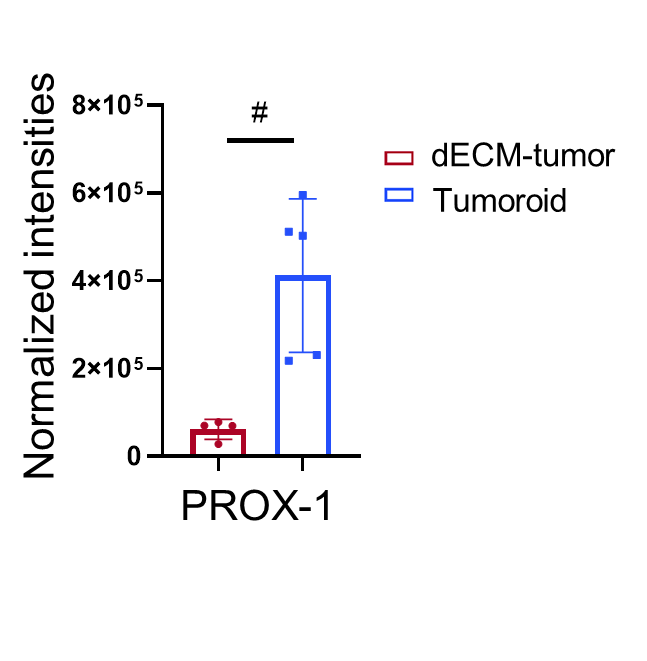


**Supplementary figure 17.** **Bar graphs showing the normalized, imputed protein intensities of PROX-1 in eluate samples from dECM-tumors (left, red, *n*=4) and tumoroids (right, blue, *n*=5).** ^#^ *q*<0.05. Data are presented as means ± SD. Zero values (when the protein was not observed by MS proteomics) were replaced with imputed values as described in Methods (Proteomic Data Analysis).

**
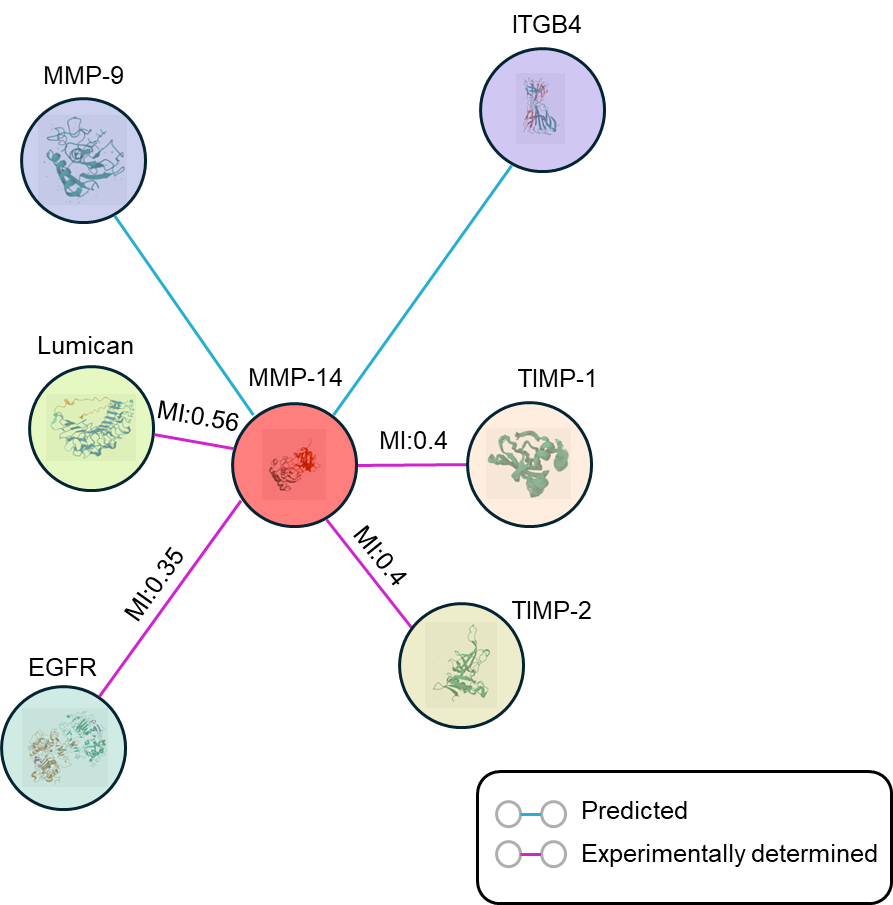
**

**Supplementary figure 18. Protein-protein physical interaction network functional enrichment analysis with the MatrixDB database.** The colors of the lines linking two proteins represent the information sources of known or predicted physical interactions. The MI scores indicate the quantitative estimates of the confidence in each experimentally determined interaction.


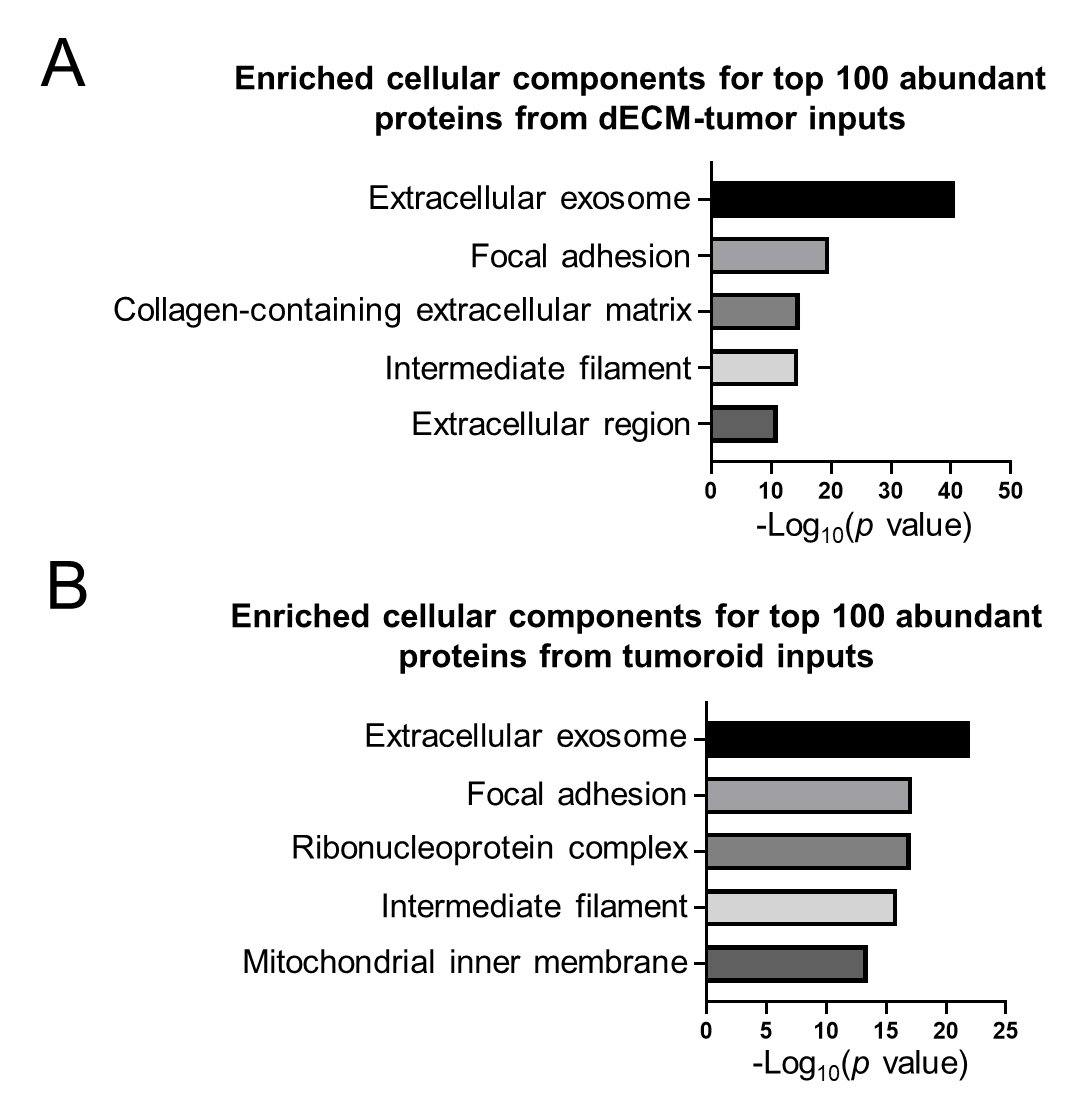


**Supplementary figure 19. GO analysis of top enriched cellular component terms of the 100 proteins with highest intensities in input samples from dECM-tumors (A) or tumoroids (B).**


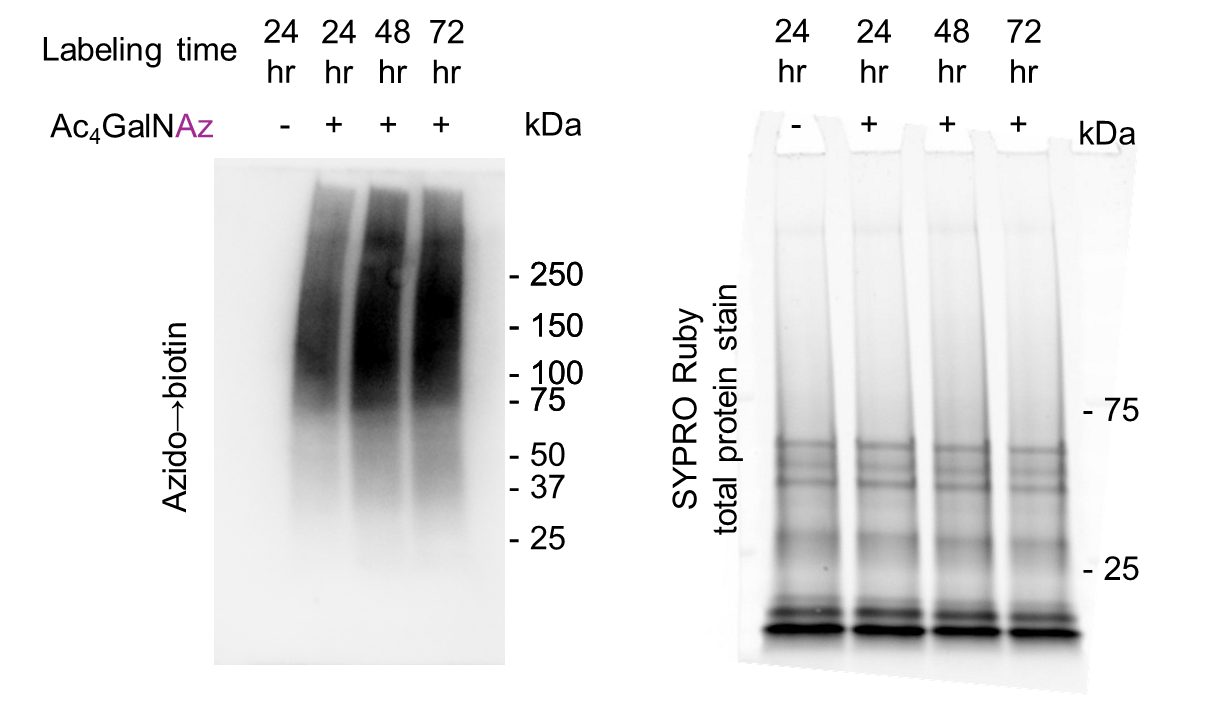


**Supplementary figure 20.** **Western blot detection of azido→biotin signal in ECM fractions of tumoroids administered with Ac_4_GalNAz for 24, 48 or 72 hours.** Azido→biotin signal detected using streptavidin-HRP (left) and SYPRO Ruby staining of total proteins (right).


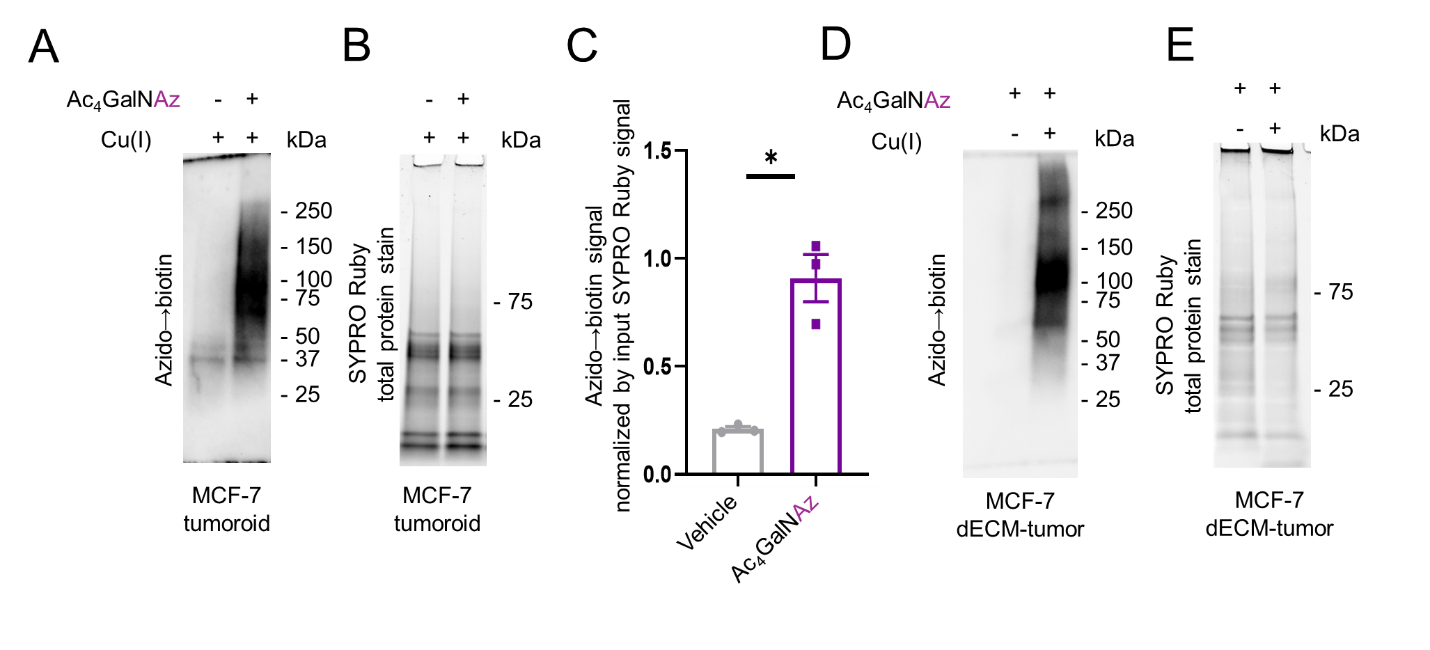


**Supplementary figure 21.** **Western blot detection of azido→biotin signal in ECM fractions of tumoroids or dECM-tumors, derived from MCF-7 cells, administered with Ac_4_GalNAz.** (A,B) Western blot detection of azido→biotin signal (A) or SYPRO Ruby staining of total proteins (B) in the ECM fractions of MCF-7 tumoroids administered with (*n*=3) or without (*n*=3) Ac_4_GalNAz. (C) Quantification of azido→biotin signals in panel A, normalized by total protein signals in panel B. The data was analyzed by two-tailed *t*-tests with Welch’s correction. (D,E) Western blot detection of azido→biotin signal (D) or SYPRO Ruby staining of total proteins (E) in the ECM fractions of MCF-7 dECM-tumor administered with Ac_4_GalNAz. The ECM fraction was reacted to alkyne-biotin with and without Cu(I) catalyst. * *p*<0.05. Data are presented as means ± SD.


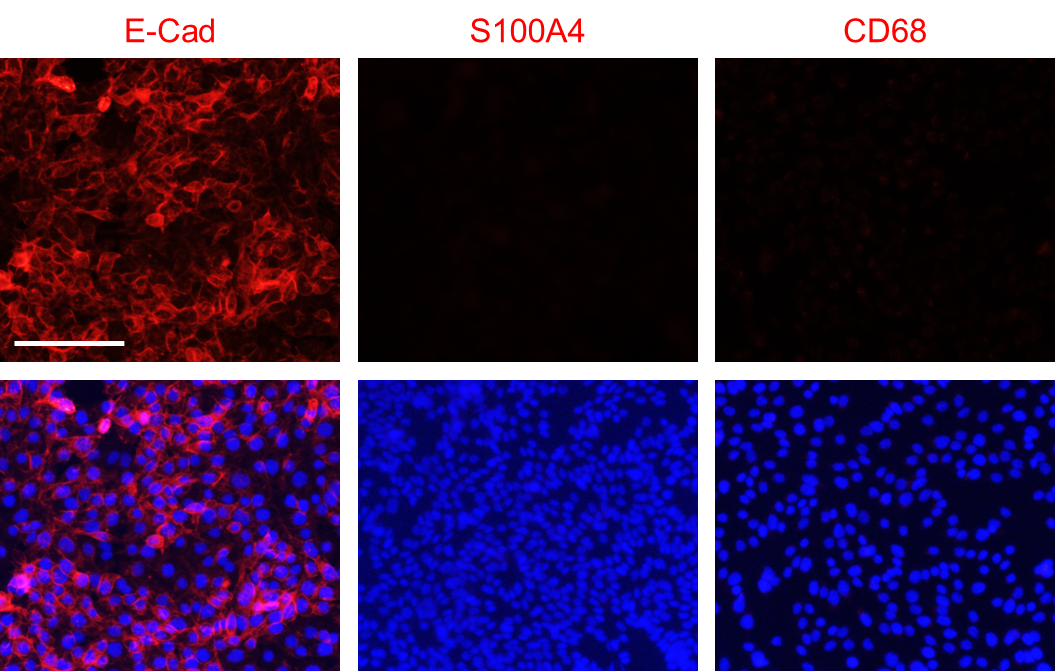


**Supplementary figure 22. Immunofluorescence staining of E-Cad (epithelial marker, red), S100A4 (fibroblast marker, red), or CD68 (monocyte and macrophage marker, red) on NCI-H358 cells cultured in a 96-well plate.** Scale bar, 150 µm.


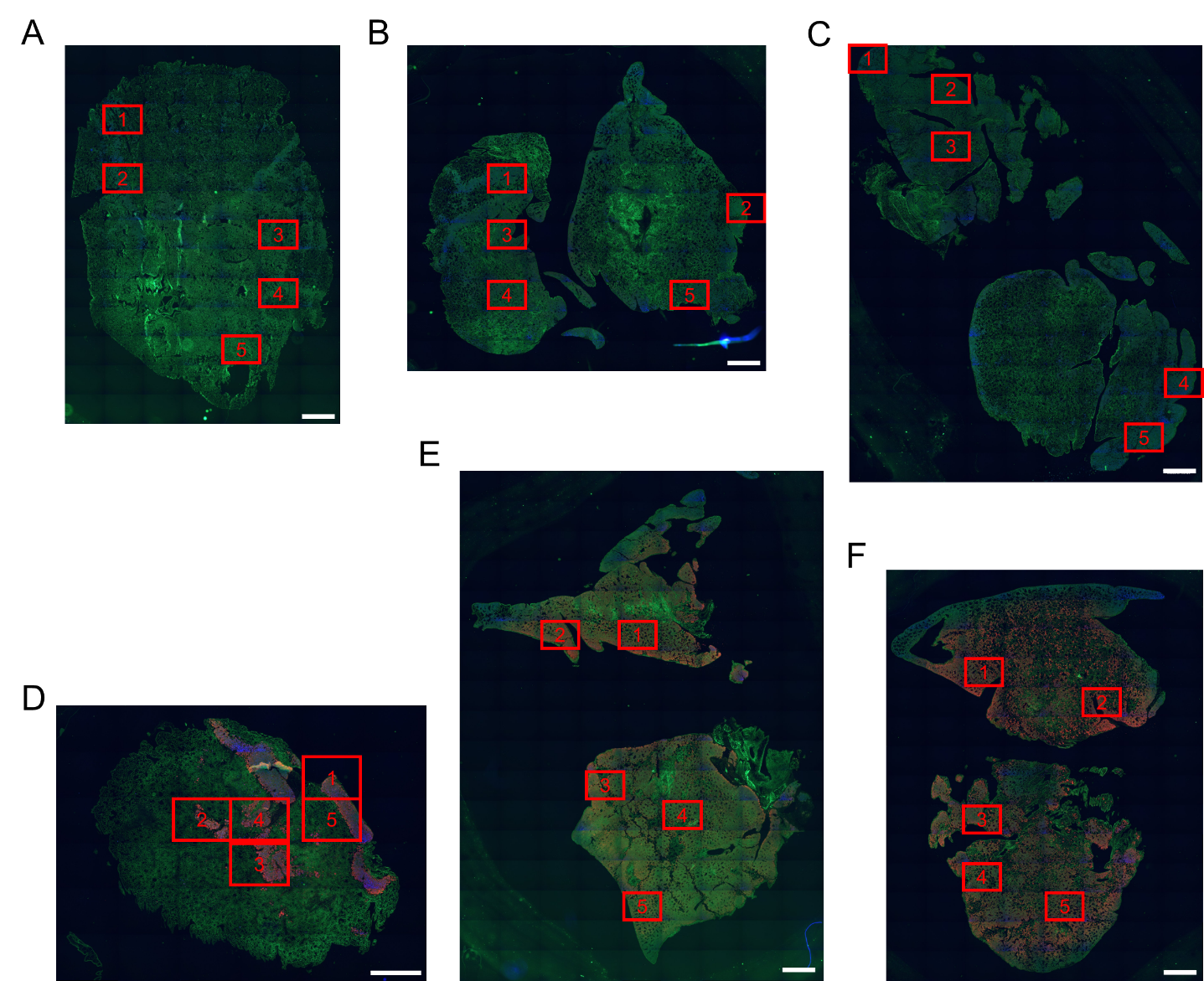


**Supplementary figure 23. Stitched images of immunofluorescence staining of azido→biotin (red) and LAMA1 (green) on dECM-tumors receiving Ac_4_GalNAz (n=3) or DMSO (vehicle control, n=3) during the last day of culture.** (A-C) Stitched images from dECM-tumors receiving DMSO. (D-F) Stitched images from dECM-tumors receiving Ac4GalNAz. Red rectangles indicate the areas selected for quantifications of azido→biotin normalized by DAPI covered areas. Five randomly-selected areas containing tumor cell clusters (DAPI positive) were analyzed for each biological replicate. Scale bar, 1000 µm.

**Table 1. Intracellular marker intensities in proportion to total human proteins identified in dECM-tumor or tumoroid inputs.**

| **Intracellular markers** | **Proportional to total human protein intensities**  **identified in inputs** | |
| --- | --- | --- |
|  | **dECM-tumor** | **Tumoroid** |
| Alpha actins | Not found | Not found |
| Beta actins | Not found | Not found |
| Gamma actins | Not found | Not found |
| Alpha tubulins | 0.50‰ | 0.77‰ |
| Beta tubulins | 1.16‰ | 1.16‰ |
| Gamma tubulins | 0.26‰ | 0.12‰ |
| Histones H1/H5 | 0.95‰ | 4.80‰ |
| Histone H2A | 0.21‰ | 0.50‰ |
| Histone H2B | 0.37‰ | 0.67‰ |
| Histone H3 | 0.14‰ | 0.12‰ |
| Histone H4 | Not found | Not found |
| Total | 0.36% | 0.81% |

**Table 2.** **Functional annotation clustering of proteins with top 100 abundance from dECM-tumor newsECM.**

| **GO category** | **GO term** | **Count** | ***p* value** | **Adjusted *p* value^[[1]](#footnote-1)^** |
| --- | --- | --- | --- | --- |
| Annotation Cluster 1 (Enrichment score: 12.41) | | | | |
| KEGG_PATHWAY | ECM-receptor interaction | 18 | 1.90E-19 | 2.60E-17 |
| GOTERM_BP_DIRECT | Cell migration | 17 | 4.40E-13 | 2.50E-10 |
| KEGG_PATHWAY | Proteoglycans in cancer | 12 | 6.70E-07 | 1.50E-05 |
| Annotation Cluster 2 (Enrichment score: 10.48) | | | | |
| GOTERM_CC_DIRECT | Collagen-containing extracellular matrix | 21 | 6.40E-15 | 5.40E-13 |
| GOTERM_CC_DIRECT | Basement membrane | 10 | 1.00E-09 | 3.10E-08 |
| UP_KW_CELLULAR_  COMPONENT | Extracellular matrix | 14 | 5.70E-09 | 6.00E-08 |
| Annotation Cluster 3 (Enrichment score: 9.61) | | | | |
| UP_SEQ_FEATURE | CARBOHYD:N-linked (GlcNAc...) asparagine | 57 | 9.10E-14 | 1.20E-10 |
| UP_KW_DOMAIN | Signal | 59 | 2.50E-10 | 5.40E-09 |
| UP_KW_PTM | Disulfide bond | 52 | 6.70E-07 | 4.50E-06 |
| Annotation Cluster 4 (Enrichment Score: 8.99) | | | | |
| UP_KW_PTM | Proteoglycan | 14 | 1.70E-13 | 2.30E-12 |
| UP_KW_PTM | Heparan sulfate | 7 | 5.40E-09 | 4.90E-08 |
| GOTERM_CC_DIRECT | lysosomal lumen | 9 | 2.50E-08 | 5.90E-07 |
| GOTERM_CC_DIRECT | Golgi lumen | 9 | 5.00E-08 | 1.00E-06 |

**Table 3. MetaMorpheus modifications for G-PTM-D.**


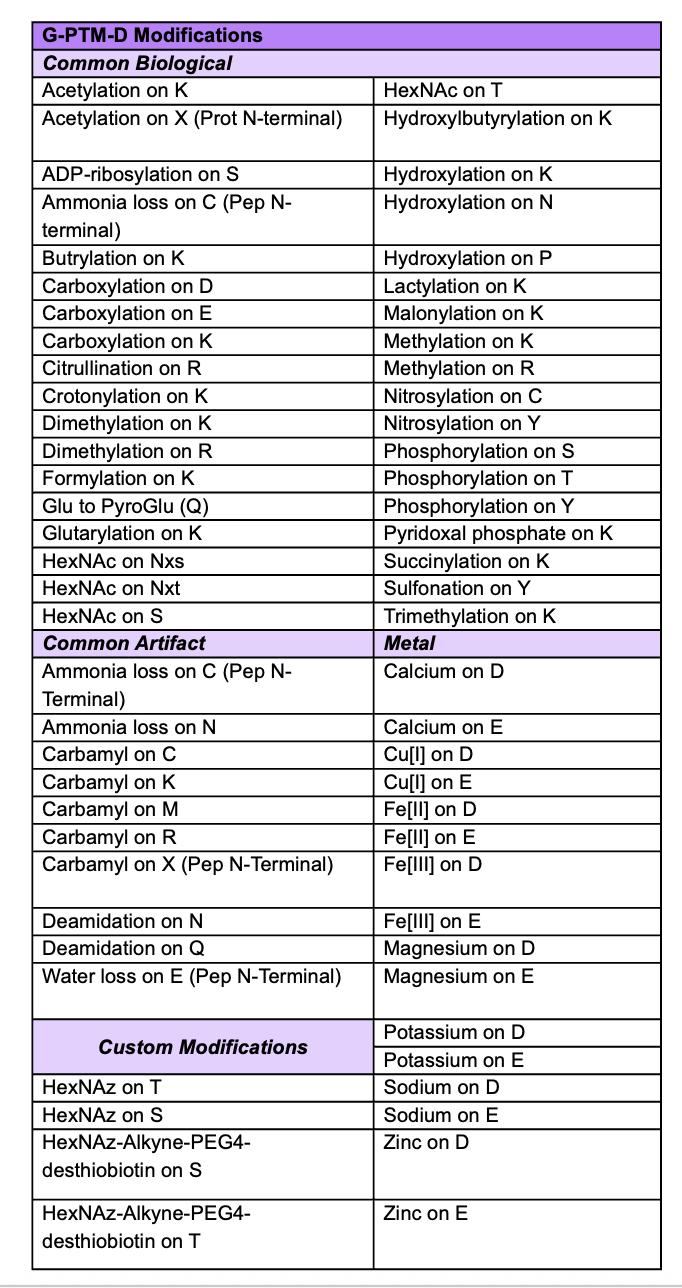


**Table 4.** **Individual protein intensities before or/and after normalization in all three searches.**

“*Search 1* original”: **original** 17 eluates (1 outliner removed) files that underwent GPTMD and were searched using the Human XML database with MBR. Column A-C: Uniport accession numbers; Gene names; Protein full names. Column D-F: 3 replicates of dECM-tumor eluates administered with DMSO. Column G-J: 4 replicates of dECM-tumor eluates administered with Ac_4_GalNAz. Column K-O: 5 replicates of tumoroid eluates administered with DMSO. Column P-T: 5 replicates of tumoroid eluates administered with Ac_4_GalNAz.

“*Search 1* normalization 1”: for comparison between Ac_4_GalNAz and Vehicle groups, the protein intensities were **normalized** separately in each treatment group. Column A-C and column M-O: Uniport accession numbers; Gene names; Protein full names. Column D-F: 3 replicates of dECM-tumor eluates administered with DMSO. Column G-J: 4 replicates of dECM-tumor eluates administered with Ac_4_GalNAz. Column P-T: 5 replicates of tumoroid eluates administered with DMSO. Column U-Y: 5 replicates of tumoroid eluates administered with Ac_4_GalNAz.

“*Search 1* normalization 2”: proteins from 9 Ac_4_GalNAz dECM-tumor and tumoroid eluate samples were **normalized** all together for their proportional intensities in each group. Column A-C: Uniport accession numbers; Gene names; Protein full names. Column D-G: 4 replicates of dECM-tumor eluates administered with Ac_4_GalNAz. Column H-L: 5 replicates of tumoroid eluates administered with Ac_4_GalNAz.

“*Search 2* original”: **original** 15 dECM-tumor files (inputs and eluates, 1 eluate removed) that underwent GPTMD analysis and were searched using the Human and Rat XML databases without MBR. Column A-C: Uniport accession numbers; Gene names; Protein full names. Column D-K: 8 dECM-tumor inputs. Column L-R: 7 dECM-tumor eluates.

“*Search 3* original”: **original** 18 dECM-tumor and tumoroid input files that underwent GPTMD analysis and were searched using the Human and Rat XML databases with MBR. Column A-D: Uniport accession numbers; Gene names; Organisms; Protein full names. Column E-N: 10 tumoroid inputs. Column O-V: 8 dECM-tumor inputs.

“*Search 3* normalization”: all input samples were **normalized** together. Column A-D: Uniport accession numbers; Gene names; Organisms; Protein full names. Column E-N: 10 tumoroid inputs. Column O-V: 8 dECM-tumor inputs.

The normalization formulas can be found under “Proteomic Data Analysis” in the Method section. The table contents are in a separate excel file.

1. *p* values were adjusted by Benjamini correction with FDR<0.05. [↑](#footnote-ref-1)
